# Postbiotics in Food Systems: Components, Production Methods, Food Applications, Functional Properties, and Technological Challenges

**DOI:** 10.1002/fsn3.72173

**Published:** 2026-07-28

**Authors:** Tansu Taspinar, Nuray Güzeler

**Affiliations:** ^1^ Department of Food Engineering, Faculty of Engineering Cukurova University Adana Türkiye

**Keywords:** food applications, functional properties, health effects, postbiotics, technological challenges

## Abstract

The functional food industry has increasingly focused on postbiotics due to challenges associated with maintaining probiotic viability and safety concerns in immunocompromised individuals. Postbiotics are defined as inanimate microbial cells and/or their metabolites that do not require viability but retain health promoting properties and technological stability. This stability has positioned postbiotics as promising alternative ingredients for food engineering applications where processing robustness is critical. This review provides a comprehensive, engineering‐oriented evaluation of postbiotics as next‐generation functional food ingredients. Current definitions and classification schemes are first summarized, followed by an assessment of how innovative thermal and non‐thermal production methods can be integrated into food processing systems. Analytical and advanced characterization techniques used for the qualitative and quantitative assessment of postbiotics in food matrices are then examined, alongside a comparison of the technological advantages and safety profiles of postbiotics relative to probiotics. Applications across different food matrices are reviewed and summarized in detailed tables, and commercially available postbiotic products are compiled together with their key specifications. Major challenges limiting broader industrial adoption are also addressed, including interactions with food components, the absence of standardized inactivation protocols, regulatory uncertainties, and the lack of harmonized industrial‐scale production methods. By consolidating these dimensions, this review identifies key research gaps, particularly the need for standardized inactivation and characterization protocols that can support regulatory alignment and evidence‐based health claims and outlines future directions to improve process efficiency and support the development of safe, sustainable postbiotic‐based foods.

## Graphical abstract

This review provides a comprehensive, engineering‐oriented synthesis of postbiotics as next‐generation functional food ingredients. It first outlines the principal production routes for postbiotic generation, encompassing thermal technologies, non‐thermal technologies, and extraction/purification methods, and subsequently examines the analytical and instrumental approaches employed for the identification and enumeration of postbiotic preparations, including viable plate counting, flow cytometry, polymerase chain reaction (PCR), and instrumental analysis techniques. Building on this technological foundation, the review consolidates current knowledge on the application of postbiotics across multiple sectors, namely food applications (dairy products, fruit and vegetable products, cereal products, meat and fish products, and food packaging), human health applications (pharmaceutical and cosmetic uses), and animal health applications (veterinary use). By integrating production, characterization, and application perspectives within a single conceptual framework, this review identifies critical research gaps, particularly the lack of standardized inactivation and characterization protocols, and highlights future directions to support the development of safe, efficacious, and industrially scalable postbiotic‐based products.

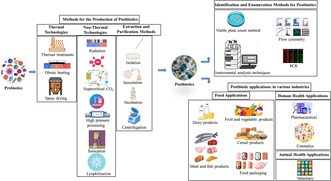

## 1 Introduction

The concept of probiotics has been defined by the FAO and WHO as “live microorganisms that, when administered in adequate amounts, confer a health benefit on the host” (Hill et al. 2014). For decades, this definition shaped the design of functional foods, placing bacterial viability at the center of product development strategies. However, maintaining microbial viability throughout food processing, storage, and gastrointestinal transit remains a persistent technological challenge, one that has constrained both product diversity and industrial scalability. Key obstacles include selecting appropriate delivery formats, preserving cell integrity under thermal, mechanical, and osmotic stresses inherent to food manufacturing, and guaranteeing functional stability through the shelf life of the final product (Awad et al. 2025; Calvanese et al. 2025; Divsalar et al. 2025; Pimentel et al. 2023).

**FIGURE 1:**
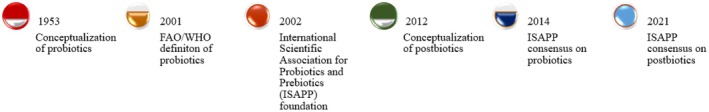
Timeline of the evolution of probiotics and postbiotics concepts.

Recent mechanistic evidence has fundamentally unsettled the viability centered paradigm. Comparative studies of live and inactivated microorganisms, and of discrete microbial fractions derived from them, have demonstrated that some beneficial effects may be retained after inactivation (Cuevas‐González et al. 2020). However, it is crucial to acknowledge that the vast majority of the evidence supporting this non‐viable equivalence is currently derived from in vitro assays and animal models. Because fundamental differences exist between human and animal physiology, these pre‐clinical findings cannot always be directly translated to human outcomes, underscoring the critical need for large‐scale, well‐designed clinical trials to validate these therapeutic effects in human populations (Calvanese et al. 2025; Ding et al. 2026; Hernández‐Granados and Franco‐Robles 2020; Mokashe et al. 2026). Cell wall components, metabolites, and other structural or secreted elements appear sufficient to mediate immunomodulatory, barrier protective, and antimicrobial responses, decoupling health functionality from the fragile requirement of cellular survival. This conceptual shift gave rise to the term postbiotic, formally defined by the *International Scientific Association for Probiotics and Prebiotics* (ISAPP) as “preparations of inanimate microorganisms and/or their components that confer a health benefit on the host” (Aguilar‐Toalá et al. 2018; Salminen et al. 2021). A timeline depicting key milestones in the field since the conceptualization of probiotics is provided in Figure 1.

Despite the growing scientific interest in postbiotics, a critical gap persists between conceptual advances and practical application. The food engineering dimensions of postbiotics remain systematically underexplored. How inanimate microbial preparations interact with complex food matrices, influencing texture, a_w_, lipid oxidation, protein conformation, and sensory properties, has not been comprehensively characterized. Equally, the field lacks standardized frameworks for postbiotic characterization, validated bioactivity markers, and harmonized regulatory definitions that would underpin consistent industrial translation. Without these foundations, reproducibility across production batches, meaningful quality benchmarking, and credible label claims remain unresolved challenges that limit the broader adoption of postbiotics in the food industry. This review therefore addresses a timely and strategically important knowledge gap at the intersection of postbiotic science, food engineering, and industrial standardization. It aims to provide a rigorous scientific basis for the applicability of postbiotics in food systems by evaluating their compositional diversity, inactivation and production methods, analytical identification tools, health associated mechanisms, and critically their demonstrated and prospective uses in food products.

## 2 Postbiotic

The ISAPP definition of postbiotics carries important implications for how these preparations must be developed and characterized. Specifically, it is emphasized that postbiotics must be derived from known and well‐characterized microorganisms, and that unidentified microorganisms should not be used for the purpose of postbiotic production. Postbiotic preparations should meet the molecular characteristics of non‐viable and inactivated microorganisms of known origin and should be beneficial to host health. Therefore, the composition and safety evaluations of postbiotic preparations are aspects that must be carefully determined and transparently reported (Wei et al. 2024). While *Lactobacillus* and *Bifidobacterium* constitute the predominant sources, postbiotics originating from *Streptococcus* and *Faecalibacterium* species have also been documented in the literature (Gurunathan et al. 2024; Prajapati et al. 2023).

**FIGURE 2:**
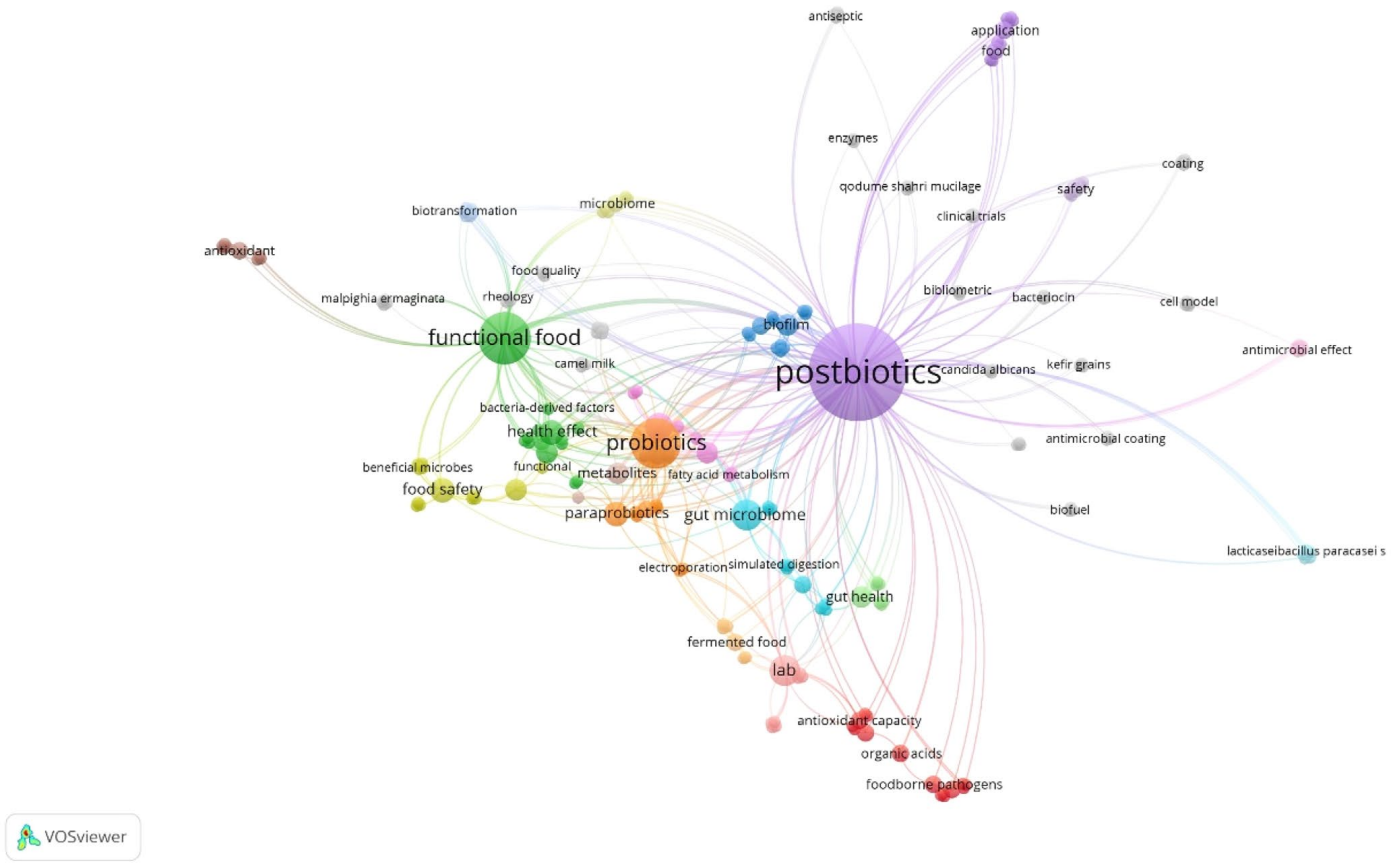
Bibliometric visualization of global research on postbiotics in food science. Created in VOSviewer (https://www.vosviewer.com/).

The co‐occurrence network generated using VOSviewer (Figure 2) was constructed from the author and index keywords of 77 documents published between 2020 and 2026, retrieved from the Scopus database using “postbiotic” as the primary search term. Keyword occurrence values reported below reflect combined author and index keyword frequencies extracted from the Scopus dataset; terms for which occurrence could not be independently verified from the source data are reported qualitatively. The exponential growth trajectory of the literature, from 3 publications in 2020 to 6 in 2021, 10 in 2022, 10 in 2023, 20 in 2024, and 25 in 2025, with an additional 3 recorded in early 2026, representing a more than eightfold increase over 5 years, underscores the rapid consolidation of postbiotics as a distinct research domain. The term *postbiotic* itself emerged as the most frequently occurring keyword cluster (combined occurrence = 101), followed by *probiotics* (*n* = 47), *functional food* (*n* = 38), *metabolites* (*n* = 21), *food microbiology* (*n* = 16), *fermentation* (*n* = 14), *lactic acid bacteria* (*n* = 13), and *gut microbiota* (*n* = 12), reflecting the dual anchoring of the field in microbial science and applied food research.

The visualization reveals the relationship of postbiotics with food engineering, food technology, and biotechnology, as well as the conceptual interactions within these fields. Therefore, the current map demonstrates that the disciplinary orientation of the literature has expanded toward an applied and industrial dimension. Terms located in the purple cluster, such as *application* (*n* = 2), *encapsulation* (*n* = 2), *cell‐free supernatant* (*n* = 5), *biopreservation* (*n* = 3), *spray drying*, *stability*, *storage*, *antimicrobial effect*, and *bacteriocin* indicate that postbiotics are being addressed in the context of food technology and biotechnological applications. The co‐occurrence of encapsulation techniques with shelf life related terms (*n* = 6) reveals that research is actively investigating the integration of postbiotics into functional products and the optimization of product longevity, suggesting that the field is pivoting from biological mechanisms toward product development and industrial feasibility. Concepts in the green cluster, including *functional food* (*n* = 38), *food safety* (*n* = 15), *bioactive compounds* (*n* = 4), *microbiome* (*n* = 5), *beneficial microbes*, and *regulation* demonstrate that postbiotics are being positioned as functional food ingredients and evaluated within food safety and regulatory frameworks. This finding implies that the field is expanding from a nutraceutical perspective, with consumer health and regulatory dimensions occupying increasing space in the literature. Meanwhile, terms in the blue cluster, such as *probiotics* (*n* = 47), *lactic acid bacteria* (*n* = 13), *fermentation* (*n* = 14), *gut microbiome* (*n* = 12), *paraprobiotics* and *fermented foods* point to the microbial origin of postbiotics and their biological continuity with probiotics. The examination of production and mechanisms of action, particularly through lactic acid bacteria (LAB) and fermentation processes, reflects the development of the field based on microbial biotechnology. Keywords in the red cluster, such as *antioxidant activity* (*n* = 4), *foodborne pathogens* (*n* = 2), *oxidative stress* and in vitro show that the antioxidant and antimicrobial effects of postbiotics are being evaluated through experimental studies. The prominence of the term in vitro indicates that a significant portion of the literature remains laboratory based and focused on mechanistic validation. Terms in the yellow cluster, such as *enzymes* (*n* = 2), *biofilm* (*n* = 4), and *fermentation* reflect research related to production processes, microbial metabolism, and enzymatic activities. This highlights the strengthening of the bioprocess engineering and microbial technology dimensions of the field. Overall, this network map reveals that postbiotic research has evolved from an initial biomedically focused phase toward stages of application, production technology, and functional product development. The relative scarcity of clinical disease terms, combined with the high occurrence of food science and biotechnology keywords, confirms that the analyzed literature is predominantly centered on applied food research. Consequently, postbiotics represent a research field in active transition, moving from a purely mechanistic subject toward translational and industrial integration, with high commercialization potential and a literature base that is currently consolidating at a rapid pace.

## 3 Methods for the Production of Postbiotics

**FIGURE 3:**
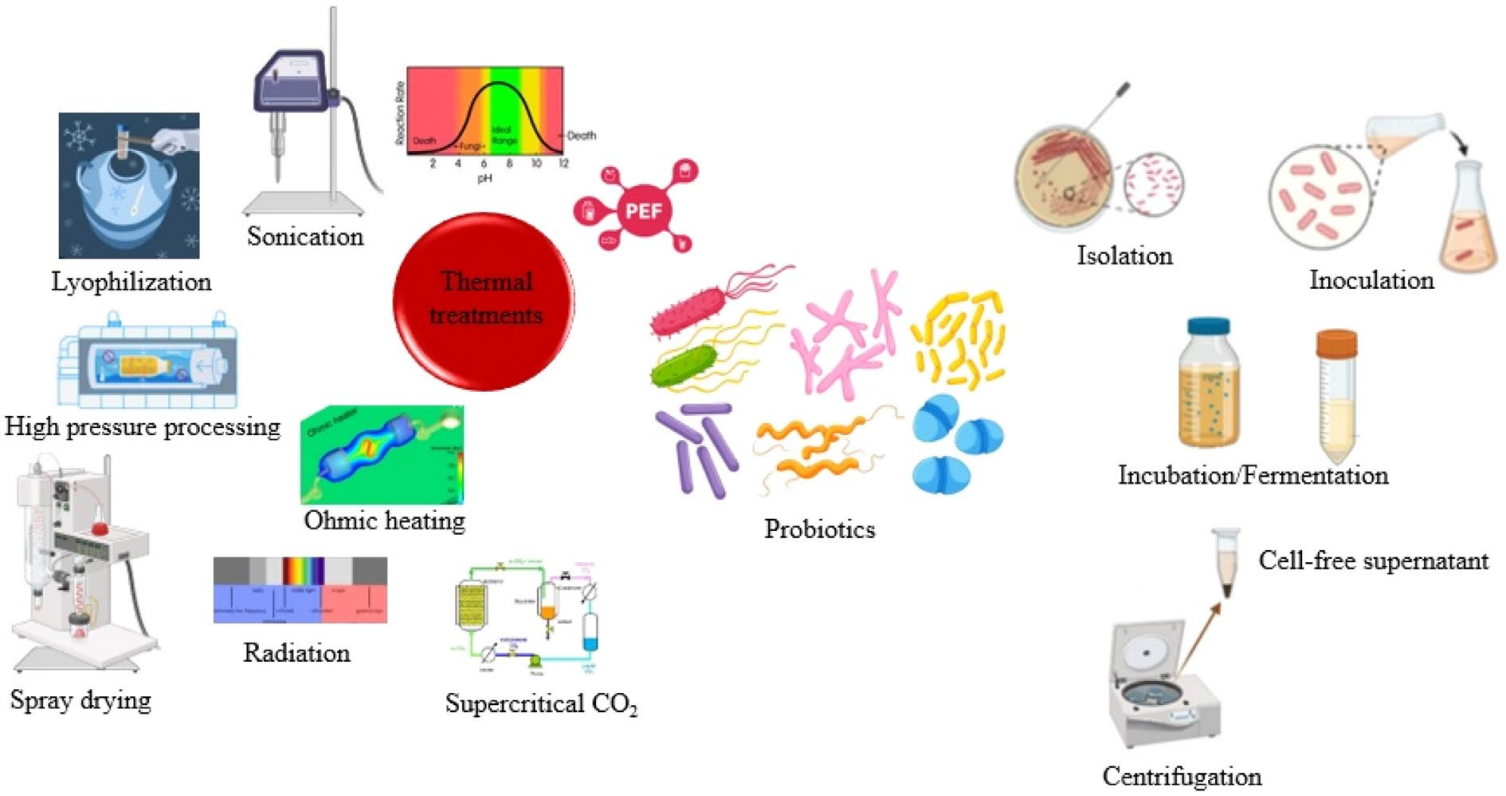
Methods used in the production of postbiotics.

Postbiotic production is a process that can be achieved using various methods and shows considerable variation across different applications. It is emphasized that the inactivation method used in postbiotic production should be capable of preserving the health promoting effects provided by probiotic microorganisms (de Almada et al. 2016). Postbiotics can be obtained through the inactivation of probiotic microorganisms using chemical methods or physical processes, each of which operates via different mechanisms (Mudaliar et al. 2024; Pimentel et al. 2023). These methods can be implemented through various mechanisms, including disruption of the cell membrane, damage to nucleic acids and DNA, protein denaturation, chemical modifications, enzyme inactivation, and protein coagulation (Lee et al. 2023). Figure 3 presents a systematic and holistic framework for two fundamental approaches to obtaining postbiotics: (i) the microorganism based fermentative production process and (ii) production strategies based on physical/thermal inactivation and advanced processing technologies. This holistic structure emphasizes that postbiotics should be evaluated not merely as biological fermentation products, but also as bioactive components obtained and stabilized through process engineering applications. The steps located in the right section represent the production of postbiotics via controlled microbial fermentation (Garg et al. 2026). In this process, metabolites like short chain fatty acids, organic acids, bacteriocins, enzymes, and bioactive peptides formed by culturing probiotic microorganisms are separated from the cellular structure and evaluated as postbiotic components (Amobonye et al. 2025; Nataraj et al. 2020). The supernatant fraction remaining after the centrifugation step or filtration represents the fraction where biologically active molecules are concentrated, providing considerable benefits with respect to controlled production, standardization, and content reproducibility (Amobonye et al. 2025; Garg et al. 2026; Nataraj et al. 2020). The techniques located in the left section represent advanced processing technologies used for microorganism inactivation or the stabilization of bioactive components in postbiotic production (Cuevas‐González et al. 2020; Lee et al. 2023). These methods aim to terminate the metabolic activity of live cells while preserving cell wall components, intracellular metabolites, and other bioactive molecules (Cuevas‐González et al. 2020; de Almada et al. 2016). Specifically, thermal and non‐thermal technologies enhance product safety by ensuring controlled inactivation (Karabacak Aydin et al. 2026; Lee et al. 2023), while thermal processing is widespread; techniques like spray drying and lyophilization play a critical role in terms of shelf life, stability, and industrial applicability (Calvanese et al. 2025; Divsalar et al. 2025; Garg et al. 2026). Furthermore, advanced extraction methods such as supercritical CO_2_ allow for the selective recovery of specific bioactive fractions (Homayouni‐Rad et al. 2025; Thirumdas and Mudgil 2025). Overall, Figure 3 demonstrates that food process engineering plays an integrated role in postbiotic production, offering a holistic production perspective that evaluates both the biological and technological dimensions of the field.

### 3.1 Thermal Treatments

It is the most commonly used method, as it involves applying heat to bacteria at a specific temperature for a defined period, is well established for microbial inactivation, and requires relatively low capital investment (Mudaliar et al. 2024). The thermal treatment to be applied is determined by taking into account the heat resistance of microorganisms. Thermal treatment is influenced by numerous factors, including the type of microorganism, whether the target cells are vegetative cells or spores, microbial growth phase, growth medium, temperature, water activity (a_w_), prior exposure to stress, the heating method, and pH. Generally, the mechanism of heat inactivation involves rupture of DNA filaments, membrane damage, ribosome aggregation, loss of nutrients and ions, protein coagulation, and inactivation of essential enzymes. Consequently, nearly all cellular structures are affected by high temperatures. It has been reported that thermal treatments increase cell surface roughness and rigidity, which in turn influence immunomodulatory properties, and that these characteristics increase in parallel with rising temperatures. The literature reports considerable variability in the conditions applied for heat inactivation of probiotics. Although time–temperature combinations vary depending on the probiotic strain and the intended health benefit, treatments are generally applied within the range of 60°C–121°C for 5 to 60 min (de Almada et al. 2016; Kawase et al. 2012). Ongoing research aims to compare the extent and duration of inactivation with the retained health promoting activity (Mudaliar et al. 2024).

### 3.2 Ionizing Radiation

The irradiation process relies on X‐ray, electron beam, or gamma ray exposure to achieve microbial inactivation. Regarding inactivation efficiency, no significant difference exists among these three distinct radiation sources. The required irradiation dose depends on the intrinsic resistance of the target organism, as well as several other factors including the radiation source, penetration depth, established safety limits, the cellular components to be preserved, and the intended application of the resulting postbiotic (Farkas 2006; Mudaliar et al. 2024; Porfiri et al. 2022; Song et al. 2016). Ionizing radiation achieves cellular inactivation primarily through the induction of damage to nucleic acids (Lee et al. 2023; Mañas and Pagán 2005). Despite its efficacy, the widespread industrial application of irradiation for postbiotic production is hindered by several limitations. From a market standpoint, the technology faces challenges regarding consumer acceptability due to negative public perceptions. Furthermore, its use is subject to strict legal restrictions, regulatory oversight, and mandatory labeling requirements that vary significantly across different countries (Thirumdas and Mudgil 2025). Technical and economic hurdles also exist, including the risk of oxidation and structural alteration of sensitive postbiotic compounds or nutrients caused by reactive oxygen species (ROS) upon excessive exposure (Mudaliar et al. 2024; Thirumdas and Mudgil 2025) as well as the high equipment and infrastructure costs required for large scale irradiation facilities (Thirumdas and Mudgil 2025).

### 3.3 Ultraviolet Radiation

Ultraviolet (UV) radiation is a non‐ionizing form of radiation characterized by its germicidal properties. UV application is highly effective in inactivating a broad spectrum of microorganisms while simultaneously preserving their beneficial immunomodulatory characteristics. Generally, the UV inactivation process involves exposing probiotics suspended in a culture medium to a UV source emitting light at a specific wavelength for a predetermined duration (Lopez et al. 2008; Mudaliar et al. 2024). The most potent microbial inactivation effects are observed within the short wavelength UV region (Lee et al. 2023). Being a non‐thermal technique, UV radiation preserves key cell wall constituents, namely lipoteichoic acids and peptidoglycans, that play a role in modulating immune responses through interaction with immune cells. The principal mechanism underlying UV‐induced bacterial lethality is generally understood to involve DNA damage. Specifically, probiotic inactivation results from the formation of pyrimidine dimers between adjacent pyrimidine bases within the same DNA or RNA strand, a process that interferes with transcription and translation and ultimately culminates in mutagenesis and cell death. Additional lethal or sub‐lethal effects encompass the formation of reactive oxygen species (ROS) that interact with DNA and cellular proteins, denaturation of proteins, and generation of DNA photoproducts (de Almada et al. 2016; Franz et al. 2009; Gomes et al. 2005; Mudaliar et al. 2024). Furthermore, damage to the cell membrane may alter membrane permeability and molecular transport processes, further contributing to cellular inactivation. Techniques such as thermal treatment, ultrasound, and ionizing radiation can be combined with UV radiation to exert synergistic effects, thereby enhancing overall inactivation efficiency (Gayán et al. 2012, 2014; Mudaliar et al. 2024; Wang et al. 2020). However, a major limitation of this technology is its restricted penetration capacity, meaning the issue of maximum UV penetration into turbid or opaque foods must be carefully addressed (Balthazar et al. 2022). Because UV light predominantly acts at the surface level, matrix‐based limitations are a critical factor to consider, especially when dealing with opaque liquids and solid foods where the penetration depth is significantly hindered (Balthazar et al. 2022; Mudaliar et al. 2024).

### 3.4 High Pressure Processing

High pressure processing (HPP) is a novel and emerging technology that involves subjecting a product to elevated pressure levels for varying durations (Chawla et al. 2011; de Almada et al. 2016). Within this technology, it is important to differentiate between high hydrostatic pressure and high pressure homogenization. High hydrostatic pressure uses a fluid medium (typically water) to transfer pressures ranging from 100 to 1200 MPa, and can be applied to both liquid and solid foods. Conversely, high pressure homogenization is a continuous or dynamic process that uses a high pressure homogenizer to apply pressures ranging from 30 to 350 MPa. This method inactivates microbes through cell ruptures caused by a combination of pressure increases and shear stress, and it is primarily applied to liquid foods and emulsions (de Almada et al. 2016). Both the applied pressure range and the duration of treatment are of critical importance in this technique. Depending on the pressure magnitude and processing time, distinct effects on cellular integrity are observed (Diels and Michiels 2006; Mudaliar et al. 2024). Within the 20–180 MPa range, microbial growth is retarded and protein synthesis is inhibited (Lado and Yousef 2002). High pressure inactivates microorganisms by inducing cell membrane damage, reducing intracellular pH, and causing protein denaturation, which collectively lead to the excessive loss of cellular constituents and the coagulation of nucleic acids and ribosomes. Furthermore, high pressure application can be coupled with thermal treatment to enhance the efficiency of cellular inactivation (Lee et al. 2023; Mañas and Pagán 2005).

### 3.5 Sonication

Sonication is a physical method that utilizes ultrasonic waves to disrupt intermolecular interactions. Ultrasound is defined as sound waves with frequencies exceeding the threshold of human perception (> 16 kHz). This method generates gas bubbles within a liquid medium; the subsequent implosion of these bubbles results in localized increases in temperature and pressure (Butz and Tauscher 2002; de Almada et al. 2016; Gibson et al. 2008). The efficacy of ultrasonic inactivation is influenced by several factors, including ultrasonic frequency and intensity, treatment duration, the nature of the target organism, temperature, and the physicochemical properties of the liquid medium (Mudaliar et al. 2024). Sonication technology brings about physical and chemical modifications in biological structures as a result of intracellular cavitation, a phenomenon that produces micro‐mechanical shocks capable of disrupting structural and functional cellular components and ultimately culminating in cell lysis. Microbial inactivation primarily occurs through the rupture of the cell wall, the disruption and thinning of the cell membrane, and DNA damage mediated by the production of free radicals (Birmpa et al. 2013; de Almada et al. 2016). Furthermore, sonication can be implemented in combination with other techniques, such as UV radiation and thermal treatment, to achieve enhanced effects (Mudaliar et al. 2024; Wang et al. 2020). However, the application of sonication at the food product level presents several notable limitations. Despite being categorized as a non‐thermal technology, the equipment used for sonication often generates bulk heat, and the implosion of cavitation bubbles produces extreme localized temperatures that can reach up to 5500°C (Amobonye et al. 2025; Mudaliar et al. 2024). This heat generation, combined with intense mechanical forces and excessive acoustic power, can destabilize heat labile metabolites, degrade sensitive bioactive compounds, and induce lipid oxidation, which may lead to undesirable off‐flavors in the final food product (Amobonye et al. 2025; Issazadeh and Hatami 2025). Furthermore, cavitation non‐uniformity is a significant challenge, as the distribution of acoustic energy can be highly uneven depending on the equipment design; for instance, ultrasound baths generally provide a less uniform energy distribution compared to probe systems (Pimentel et al. 2023). Finally, regarding energy consumption, although ultrasound can be more energy efficient than traditional thermal heating, scaling up the technology for continuous industrial processing remains difficult because maintaining the high intensity and power required for effective cell disruption across large volumes poses significant operational and energy‐related economic constraints (Thirumdas and Mudgil 2025).

### 3.6 Pulsed Electric Field

Pulsed electric field (PEF) technology represents a non‐thermal processing method that relies on delivering brief, high‐voltage electrical pulses to food products positioned between, or flowing through, a pair of electrodes (Góngora‐Nieto et al. 2004). Microbial inactivation is achieved through the disruption of the microbial cell membrane, a phenomenon known as electroporation, and typically requires an electric field intensity ranging from 2 to 90 kV/cm (de Almada et al. 2016; Devlieghere et al. 2004; Góngora‐Nieto et al. 2004). To achieve the desired levels of microbial inactivation, it is first necessary to investigate the thermal the defense mechanisms exhibited by the target microorganism, as well as elucidating the kinetics underlying PEF‐induced inactivation. Subsequently, critical PEF parameters, like pulse duration, processing time, and electric field intensity must be optimized (Álvarez et al. 2003; Mudaliar et al. 2024). In the context of postbiotics generation, PEF plays a crucial role by facilitating the release of intracellular bioactive molecules, including proteins, exopolysaccharides, and microbial metabolites through either reversible or irreversible electroporation, avoiding the extensive molecular degradation often caused by conventional thermal techniques (Thirumdas and Mudgil 2025). Furthermore, sublethal PEF applications can induce stress reactions that stimulate microbial cells to produce and release secondary metabolites (Pimentel et al. 2023). For example, sublethal PEF treatments applied to *Lacticaseibacillus rhamnosus* and *Lacticaseibacillus paracasei* have been shown to increase lactic acid production and enhance the release of proteins (Pimentel et al. 2023; Thirumdas and Mudgil 2025). Additionally, PEF treatment of *Lactococcus lactis* subsp. *cremoris* stimulated its metabolism, resulting in double the EPS yield compared to untreated samples (Thirumdas and Mudgil 2025). Other direct examples include the enhancement of β‐glucosidase enzyme activity in strains like *Lactobacillus casei* and *Lactobacillus fermentum* , which significantly improved the bioconversion of isoflavone glucosides into bioactive aglycones, as well as the increased surface binding and accumulation of calcium ions in *L. rhamnosus* cellular structures within enriched food matrices like ice cream (Balthazar et al. 2022; Thirumdas and Mudgil 2025).

### 3.7 Ohmic Heating

Ohmic heating achieves microbial inactivation through the passage of electric current directly through the target microorganisms, causing both the microorganisms and their surrounding medium to heat up due to the electrical resistance encountered. The mechanism of probiotic inactivation using ohmic heating is identical to that of conventional thermal treatments, with the additional contribution of non‐thermal damage resulting from electroporation. However, ohmic heating offers several advantages over traditional thermal processing methods, including shorter processing durations, superior preservation of the food matrix quality, and improved energy efficiency (Barros et al. 2021a; Barros et al. 2021b; Cappato et al. 2017; Mudaliar et al. 2024). Despite these advantages, the successful implementation and scaling up of ohmic heating present several challenges. A primary prerequisite is conductivity dependency, meaning the target material must be sufficiently electrically conductive, which relies heavily on its water and ionic content (Balthazar et al. 2022). Furthermore, scaling up the process is complicated by the fact that electrical conductivity increases as the temperature rises, making it difficult to precisely control the endpoint temperature and increasing the risk of overheating or underheating. These operational challenges are further exacerbated by matrix heterogeneity and equipment design; irregular shapes in treatment chambers and diverse properties within probiotic mixtures can cause local temperature variations, potentially allowing microorganisms to survive in cooler crevices (Mudaliar et al. 2024).

### 3.8 Supercritical Carbon Dioxide

Supercritical carbon dioxide (SC‐CO_2_) technology is a method in which CO_2_ is utilized above its critical point temperature and pressure values, existing in a single phase. The primary inactivation mechanism involves three key parameters: pressure, temperature, and time (de Almada et al. 2016; Efaq et al. 2015). Due to its high diffusivity and low viscosity, SC‐CO_2_ can penetrate the cell membranes of microorganisms. Such penetration compromises membrane integrity, thereby increasing permeability and triggering the release of intracellular constituents. Furthermore, dissolution of SC‐CO_2_ in aqueous media results in the formation of carbonic acid, which lowers the pH of the environment. This acidification interferes with cellular metabolic processes and negatively impacts microbial viability. Furthermore, SC‐CO_2_ facilitates the dissolution and extraction of lipids, proteins, and other vital constituents necessary for microbial survival (Amaral et al. 2017; Mudaliar et al. 2024). SC‐CO_2_ technology is characterized as a cost‐effective, non‐toxic, and environmentally friendly method (Lee et al. 2023; Yuk and Geveke 2011). However, the widespread commercial implementation of SC‐CO_2_ is often hindered by high initial investment costs and the need for highly specialized equipment and technical expertise (Thirumdas and Mudgil 2025). While it operates at relatively moderate pressures compared to high hydrostatic processing, typically requiring operating pressures between 8 and 30 MPa, the systems still demand robust and precise control (Homayouni‐Rad et al. 2025; Issazadeh and Hatami 2025; Pimentel et al. 2023). Additionally, a major limitation of this technology is its extraction selectivity because SC‐CO_2_ has very low polarity, efficiently extracting polar or moderately polar bioactive compounds is difficult and often necessitates the addition of a co‐solvent to improve polarity (Homayouni‐Rad et al. 2025; Wei et al. 2024). Finally, the extraction process can lead to potential alterations of membrane‐associated molecules, as the aggressive dissolution and removal of polar lipids and other vital constituents permanently modifies the structural and functional integrity of the microbial cell membrane (Balthazar et al. 2022; de Almada et al. 2016).

### 3.9 Dehydration

Dehydration is a process that induces microbial inhibition through the reduction of water activity (a_w_) (de Almada et al. 2016; Foerst and Kulozik 2012). This removal of free water is particularly important because liquid postbiotics with high a_w_ can promote unwanted microbial growth and are difficult to incorporate into solid food matrices in large volumes (Divsalar et al. 2025). Therefore, drying not only inhibits microbes but also serves to condense and concentrate the bioactive compounds, facilitating stability during long‐term storage (Amobonye et al. 2025; Divsalar et al. 2025). Dehydration methods primarily encompass freeze drying (lyophilization) and spray drying (Lee et al. 2023; Prapa et al. 2023).

#### 3.9.1 Lyophilization

Lyophilization is a process conducted by first freezing the target microorganisms and subsequently heating them under high vacuum, which facilitates the sublimation of water (Fu et al. 2018; Mudaliar et al. 2024). It is considered a non‐thermal approach that is highly effective at preserving the properties of biologically sensitive materials and generally offers superior product quality compared to spray drying (Amobonye et al. 2025; de Almada et al. 2016; Divsalar et al. 2025). However, it is a time‐consuming and expensive treatment (de Almada et al. 2016; Divsalar et al. 2025; Mehta et al. 2023). Furthermore, a notable drawback of lyophilization is that the extensive removal of liquid constituents can lead to the loss of essential volatile compounds, such as hydrogen peroxide, which may subsequently diminish the antimicrobial functionality and bioactivity of the resulting postbiotics (Amobonye et al. 2025; Sharafi et al. 2023; Thorakkattu et al. 2022).

#### 3.9.2 Spray Drying

In the spray drying process, a feed solution containing probiotic cells and protective agents, either dissolved or in suspension, is atomized to form fine droplets. These droplets are rapidly converted into solid particles by drying through a hot air stream with low relative humidity (Fu et al. 2018; Mudaliar et al. 2024). This method is often preferred for commercial scale‐up because it is faster and more scalable than lyophilization (Garg et al. 2026). However, exposure to high temperatures, typically ranging from 150°C to 180°C, can have deleterious effects on sensitive compounds and carries a high risk of volatilizing crucial metabolites like ethanol and acetic acid, which reduces postbiotic functionality (de Almada et al. 2016; Divsalar et al. 2025; Thorakkattu et al. 2022). During spray drying, the microorganisms are subjected to both dehydration and heat shock. These conditions induce structural alterations in nucleic acids, ribosomes, and proteins, while simultaneously leading to the degradation of the cytoplasmic membrane (Lee et al. 2023; Puttarat et al. 2021). To mitigate these issues and preserve metabolite integrity, spray drying is often combined with microencapsulation techniques to protect the bioactive components during processing and subsequent storage (Amobonye et al. 2025; Mokashe et al. 2026).

### 3.10 pH

Another method for inactivating microorganisms is the modification of the substrate's pH value. This non‐thermal approach is based on the exploitation of the minimum and maximum pH limits within which specific microorganisms can sustain growth. Alterations in intracellular pH can induce chemical modifications in essential compounds, thereby leading to microbial inactivation. Furthermore, fluctuations in pH levels can result in significant damage to the cell membrane (Aronsson and Rönner 2001; de Almada et al. 2016). The stress induced by extreme pH modifications also changes proton efflux, increases acid catabolism, and induces DNA repair enzymes. However, microbial resistance to pH modification is highly strain specific. For instance, *Lacticaseibacillus casei* exhibits strong resistance to acidic conditions and requires an extreme alkaline environment (pH 12.75) for complete inactivation. In contrast, *Lactobacillus acidophilus* can be inactivated at pH 1 for 60 min or pH 12.75 for 80 min, whereas *Bifidobacterium animalis* only requires 60 min of exposure to either of these extreme pH values (Pimentel et al. 2023). Consequently, successful pH inactivation protocols typically employ extreme ranges such as pH 1 or pH 12.5 to 12.75 applied for durations between 30 and 120 min depending on the target microorganism (Issazadeh and Hatami 2025). Beyond cellular inactivation, the pH of the surrounding matrix significantly impacts the stability and functionality of the extracted postbiotic metabolites (Divsalar et al. 2025; Mishra et al. 2024). The optimal antimicrobial activity of postbiotics is generally maintained within a pH range of 4 to 9 (Aggarwal et al. 2022; Divsalar et al. 2025; Mishra et al. 2024). Exposing these metabolites to excessively acidic or alkaline food environments can degrade sensitive compounds and drastically reduce their effectiveness (Divsalar et al. 2025; Mishra et al. 2024). For example, artificially neutralizing the pH of certain postbiotic solutions has been shown to result in the complete loss of their antimicrobial activity against target pathogens (Moradi et al. 2020).

The inactivation methods described above are summarized comparatively in Table 1, which presents a structured overview of thermal treatment, HPP, UV radiation, ionizing radiation, PEF, sonication, ohmic heating, SC‐CO_2_, lyophilization, and spray drying. For each method, the table details the primary mechanism of microbial inactivation, scale‐up feasibility, relative cost, degree of bioactivity preservation, key technological limitations, and representative food industry applications. This comparative framework is intended to facilitate the selection of appropriate inactivation strategies for postbiotic production in the context of specific food systems and industrial constraints.

**TABLE 1:** Comparative overview of microbial inactivation methods for postbiotic production.

Method	Inactivation mechanism	Scale‐up feasibility	Cost	Bioactivity preservation	Key limitations	Food applications
Thermal treatment	Membrane damage, ribosome aggregation, DNA strand rupture, enzyme inactivation, protein coagulation	*High* Well‐established industry infrastructure	*Low* Low capital investment	*Moderate* Increases cell surface roughness; may enhance immunomodulatory properties at higher temps	High variability in *D*/*z*‐values across strains; all cellular structures affected; optimisation required per strain	Dairy, beverages, fermented products, bakery
Ionizing radiation	Nucleic acid damage (DNA/RNA strand breaks) as primary mechanism	*Moderate* Requires specialized, costly irradiation facilities; not yet widespread for postbiotics	*High* High equipment and infrastructure costs for large‐scale facilities	*Moderate‐High* Risk of ROS‐mediated oxidation and structural alteration of sensitive postbiotic compounds at excessive doses	Negative consumer perception; strict regulatory oversight, legal restrictions and mandatory labelling varying by country; dose depends on organism resistance, radiation source, penetration depth, safety limits and intended application	Currently limited industrial use for postbiotics; potential application across various food matrices pending regulatory and consumer acceptance
Ultraviolet radiation	Pyrimidine dimer formation in DNA/RNA; ROS generation; protein denaturation; membrane permeability alteration	*Moderate* Limited to transparent/liquid matrices	*Low‐Moderate*	*High* Preserves peptidoglycans and lipoteichoic acids; retains immunomodulatory activity	*Low* Penetration depth; limited to clear liquid media; UV dose must be precisely controlled	Clear beverages, liquid food matrices, surface decontamination
High pressure processing	Cell membrane damage, intracellular pH reduction, protein denaturation, nucleic acid and ribosome coagulation	*Moderate* Batch process; limited throughput	*Moderate‐High* High equipment cost	*High* Non‐thermal; minimal heat damage to functional components	Pressure range critical (30–350 MPa); extended processing time needed; combinable with thermal for higher efficiency	Juices, ready‐to‐eat foods, meat products, dairy
Sonication	Intracellular cavitation causing cell wall rupture, membrane thinning, DNA damage via free radicals	*Low‐Moderate* Probe‐based systems limit volume	*Moderate*	*Moderate* Cavitation may degrade some bioactive molecules	Incomplete inactivation at low intensity; heat generation; efficacy depends on medium viscosity and frequency	Liquid foods, dairy, juices; mostly combinatorial use (thermo‐sonication, UV‐sonication)
Pulsed electric field	Electroporation‐irreversible disruption of cell membrane integrity	*Moderate* Continuous‐flow systems available	*Moderate‐High* High‐voltage equipment	*High* Non‐thermal; preserves heat‐sensitive bioactive components	Requires optimisation of pulse duration, processing time, and field intensity (2–90 kV/cm) per organism; not suitable for solid foods	Juices, liquid dairy, liquid egg products
Ohmic heating	Resistive heating+electroporation (non‐thermal component); identical endpoint to conventional thermal treatment	*Moderate* Continuous‐flow designs available	*Moderate* Lower energy use than conventional thermal	*High* Faster processing; superior matrix quality preservation	Electrode corrosion; limited to electrically conductive matrices; optimisation required per food system	Particulate foods, soups, dairy, liquid whole egg
Supercritical carbon dioxide	Membrane penetration→ permeability disruption→ intracellular leakage; pH reduction via carbonic acid; lipid and protein extraction	*Low* Complex pressurized equipment; niche adoption	*Moderate* Cost‐effective vs. other novel methods; non‐toxic	*High* Non‐thermal; environmentally friendly; preserves heat‐sensitive compounds	Limited to CO_2_‐compatible matrices; high‐pressure equipment required; not yet widely adopted in food industry	Liquid foods, beverages, nutraceutical preparations
Lyophilization	Water activity reduction via freezing followed by sublimation under high vacuum	*Low* Batch process; long cycle times	*High* Energy‐intensive; high capital cost	*Very high* Excellent structural preservation; long‐term stability	Slow and costly; not suitable for continuous production; requires reconstitution for some applications	Functional food powders, nutraceuticals, dietary supplements, infant formula
Spray drying	Combined dehydration and heat shock; induces nucleic acid/ribosome/protein structural changes; cytoplasmic membrane	*High* Continuous process; large‐scale compatible	*Low‐Moderate* Cost‐effective at scale	*Moderate* Thermal stress may compromise some bioactive components; protective agents required	Heat+dehydration stress combined; requires protective carrier agents; particle size control critical	Dairy powders, instant beverages, encapsulated ingredients, functional food powders

**FIGURE 4:**
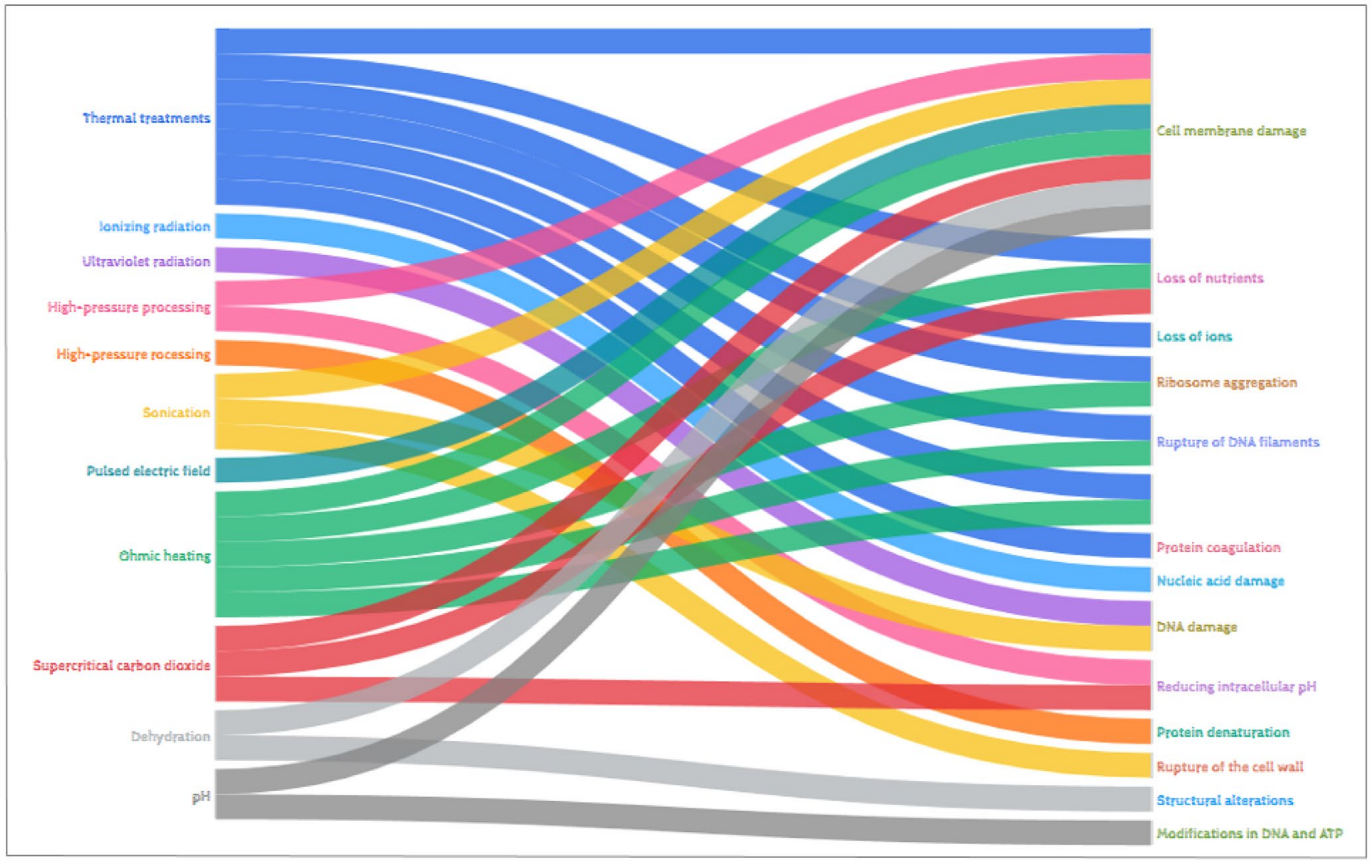
Microbial inactivation mechanisms of various technologies. Created in Flourish (https://flourish.studio/).

Figure 4 schematically illustrates the relationships between inactivation methods employed in postbiotic production and the corresponding structural and functional damage mechanisms induced in microorganisms. It demonstrates that distinct inactivation technologies exert their effects not through a single target, but rather through multiple and interrelated cellular damage mechanisms. This suggests that the processes utilized in postbiotic production aim not merely to eliminate microbial viability, but also to achieve the controlled modification of cellular components. Overall, the figure presents a comparative overview of the mechanistic foundations of inactivation strategies used in postbiotic production and underscores the critical importance of process selection regarding the targeted biological effect and product stability. This approach indicates that process parameters must be carefully optimized to obtain postbiotics that are safe, standardized, and retain their functional properties.

### 3.11 Extraction and Purification Methods

Extraction and purification methods, such as centrifugation, lyophilization, column purification, and dialysis are employed to separate bacterial cells from postbiotic metabolites within postbiotic mixtures (Cuevas‐González et al. 2020; Gurunathan et al. 2024). Centrifugation and ultrafiltration were the initially adopted methods and remain the most widely utilized techniques. In the production of postbiotics, following the disruption of the microorganism, cells undergo lysis, resulting in the liberation of intracellular metabolites, cell membrane fragments, cell wall components, and other cellular constituents (Wei et al. 2024). In order to isolate a cell‐free supernatant while ensuring the complete removal of any residual viable cells, the postbiotic mixture is subjected to centrifugation (typically at 4.000–12.000 × g for 10 min at 4°C). This is followed by the application of microfiltration and ultrafiltration techniques, after which the postbiotics are passed through a filter with a pore size ranging from 0.22 to 0.45 μm. As a final stage, the postbiotics are concentrated. For the concentration of postbiotics, lyophilization is more commonly preferred over spray drying (Moradi et al. 2021; Sharafi et al. 2023).

### 3.12 Influence of Fermentation Medium on Postbiotic Composition

The quantity, diversity and functional properties of the resulting postbiotics vary depending on the specific bacterial strain and the culture medium utilized (Homayouni‐Rad et al. 2025). The fermentation medium represents a critical factor that significantly influences the composition and functional properties of postbiotics; therefore, the selection of an appropriate medium is essential. De Man, Rogosa, and Sharpe (MRS) broth, the most frequently employed medium for LAB fermentation, has been successfully utilized for postbiotic production. However, the yellow‐brown coloration of postbiotics produced in MRS broth constitutes a limiting factor for their application in food systems, illustrating that certain methods employed in postbiotic production may adversely affect the functional properties of these compounds. Alternative media and biomass sources such as whey, modified ultrafiltered whey, whey protein, low heat treated milk, and hydrolyzed whey supplemented with soy flour have been utilized to produce postbiotics intended for use in food packaging. Nevertheless, the use of such alternatives may necessitate certain modifications and supplementations prior to microbial inoculation (İncili et al. 2023; Sharafi et al. 2023).

### 3.13 Factors Affecting Postbiotic Efficacy

A considerable body of literature indicates that both intrinsic and extrinsic factors can affect postbiotic efficacy. In particular, the active metabolites of postbiotics may interact with intrinsic factors, including the resident microbiota, enzymes, and diverse food constituents, potentially compromising their functionality; proteolytic enzymes such as pepsin, trypsin, and chymotrypsin, for instance, have been implicated in the impairment of postbiotic function. Among extrinsic factors, food processing techniques such as heat treatment, irradiation, sonication, and HPP may affect postbiotic composition due to their potential impacts on the microorganisms involved in fermentation. Temperature is a further extrinsic factor that influences the antibacterial activity of postbiotics (Aggarwal et al. 2022; Gurunathan et al. 2024).

In conclusion, inactivation parameters vary depending on the specific strain utilized, patterns of microbial resistance, the initial size of the microbial population, and environmental parameters including the composition of the growth medium; therefore, these parameters must be optimized individually for each application. The optimization of processing parameters and the identification of the specific microorganism strains that provide the desired functional benefits are of paramount importance, necessitating further research in this field.

## 4 Classification of Postbiotics

The term “postbiotic” encompasses a wide array of diverse components. According to the definition established by the ISAPP, postbiotics comprise inanimate cells and microbial metabolites, including microbial lysates, proteins, enzymes, peptides, microbial cellular components, extracellular vesicles, organic acids, bacteriocins, vitamins, exopolysaccharides, cell wall constituents, polymers, peptidoglycans, peptidoglycan derived muropeptides, teichoic acids, pili‐like structures, cell‐free extracts, cell surface fractions, culture supernatants, and biosurfactants (Gurunathan et al. 2024; Salminen et al. 2021; Taverniti and Guglielmetti 2011; Tsilingiri and Rescigno 2013).

Postbiotics can be classified according to several criteria, such as the species of the producing microorganism, their structural composition, and their physiological functions (Gurunathan et al. 2024; Malashree et al. 2019; Nataraj et al. 2020). In general terms, postbiotics may be distinguished on the basis of their chemical composition, encompassing lipid‐based compounds (e.g., propionate, butyrate, and dimethyl acetyl‐derived plasmalogens), protein‐based molecules (e.g., the p40 protein and lactocepin), carbohydrate‐based structures (e.g., teichoic acids and galactose‐rich polysaccharides), vitamins and cofactors (notably B‐group vitamins), and organic acids (e.g., 3‐phenyllactic acid and propionic acid), in addition to complex macromolecules such as peptidoglycan‐derived muropeptides and lipoteichoic acids. Alternatively, classification may be based on physiological function, encompassing anti‐inflammatory, immunomodulatory, anti‐obesity, anti‐ hypocholesterolemic, hypertensive, antioxidant, and anti‐proliferative properties (Aguilar‐Toalá et al. 2018; Nakamura et al. 2016). The classification of postbiotic components is presented in Table 2.

**TABLE 2:** Classification of postbiotic components.

Component	Source	Chemical nature	Biological role (s)	Analytical criteria	Food application suitability/examples
Inanimate cells (paraprobiotics)	Inactivated LAB and other probiotic‐associated bacteria (via thermal, UV, HPP treatment)	Whole inactivated bacterial cells; non‐viable but structurally intact	Immune system enhancement; production of beneficial metabolites (peptides, SCFAs); effects comparable to viable probiotics (“probiotic paradox”)	Confirmation of non‐viability (plate count); structural integrity assessment; comparison with viable‐strain bioactivity	Suitable where viability cannot be maintained; functional foods, dairy analogues, infant formula
*Structural components of paraprobiotics*					
Peptidoglycan (cell wall)	Cell wall of LAB	Cross‐linked glycan chains of β‐1,4‐linked N‐acetylmuramic acid and N‐acetylglucosamine	Structural integrity; immune modulation via cytokine expression	Glycan chain length analysis; cross‐linking degree; immunoassay for cytokine response	Functional ingredient for immune‐supportive foods; stable under processing
Teichoic acids	Cell wall of LAB (covalently linked to peptidoglycan or membrane‐anchored)	Anionic polymers	Immune response triggering; linked to antibiotic resistance pathophysiology	Anionic charge characterization; linkage analysis (peptidoglycan vs. membrane)	Relevant to immunomodulatory food formulations; requires careful strain selection
Cell wall polysaccharides	Bacterial cell wall (LAB)	Polysaccharide structures	Anti‐pathogenic and adhesive interactions; modulation of intestinal homeostasis and mucosal immunity	Adhesion assays; structural characterization of polysaccharide chains	Potential use in gut‐health‐targeted functional foods
Cell surface proteins	Cell envelope of LAB	Integral structural proteins	Structural and functional cell envelope integrity	Protein extraction and characterization (SDS‐PAGE, LC–MS)	Limited direct food application; primarily mechanistic relevance
Moonlighting proteins	Cytoplasm of LAB	Diverse protein group: ribosomal proteins, metabolic enzymes, elongation factors, chaperones	Multifunctional roles beyond canonical cytoplasmic function	Proteomic profiling; functional annotation	Research‐stage; not yet standardized for food use
LPxTG proteins	Cell wall of LAB (sortase A‐mediated anchoring)	Proteins with C‐terminal LPxTG motif, covalently anchored to peptidoglycan	Structural anchoring; potential adhesion‐related functions	Motif identification (LPxTG); sortase‐dependent anchoring verification	Limited current food application; mechanistic/structural interest
Pili proteins	Cell surface of *Lactobacillus* strains	Filamentous surface proteins	Bacterial motility; adherence to intestinal mucosa; persistence in the gastrointestinal tract	Microscopic visualization; adhesion assays	Relevant for designing gut‐adherent functional postbiotic preparations
S‐layer proteins	Outermost cell envelope layer of LAB (non‐covalently bound to peptidoglycan)	Self‐assembling surface protein layer	Adhesion; immune interaction	Non‐covalent binding verification; surface layer imaging (electron microscopy)	Potential carrier/adhesion‐enhancing component in functional foods
*Other postbiotic components*					
Cell‐free supernatants (CFS) and suspensions	Secreted by LAB and yeasts during fermentation	Broad category of biomolecules: organic acids, diacetyl, CO_2_, bacteriocin‐like inhibitory substances (BLIS); composition depends on growth medium	Antibacterial, anti‐biofilm, anti‐inflammatory, antioxidant, anti‐cancer activity; metabolic disorder management; diarrhea treatment	Compositional profiling (HPLC/GC–MS); bioactivity assays (antibacterial, antioxidant); medium‐dependence standardization	Widely applicable as liquid functional ingredients; medium composition (MRS broth coloration) may limit sensory acceptability
Cell wall fragments (peptidoglycan‐derived muropeptides, teichoic/lipoteichoic acids)	Released during bacterial growth, remodeling, and death	Glycan chains cross‐linked via oligopeptide bridges; immunogenic wall components	Immune system regulation; anti‐inflammatory activity via pro‐inflammatory cytokine modulation; teichoic acids linked to antibiotic resistance pathophysiology	Muropeptide profiling; cytokine expression assays; immunogenicity testing	Suitable for immune‐supportive functional food ingredients
Exopolysaccharides (EPS)	Secreted by LAB	High‐molecular‐weight extracellular carbohydrate polymers	Defense against phages/phagocytes/toxins; immunomodulatory, anti‐tumor, anti‐mutagenic, antioxidant, anti‐inflammatory, anti‐hypertensive, antibacterial, antiviral, cholesterol‐lowering, anti‐gastrointestinal activity	Recovery via centrifugation, deproteinization, filtration, dialysis, lyophilization; molecular weight determination; bioactivity panels	Texturizing and bioactive functional ingredient (fermented dairy, encapsulation matrices)
Enzymes	*Bacillus subtilis* , *Bacillus licheniformis* , *Aspergillus niger*, *Aspergillus oryzae* (primary sources)	Catalytic proteins; six classes (oxidoreductases, transferases, hydrolases, lyases, isomerases, ligases)	Catalysis of physiological, metabolic, and regulatory processes	Enzymatic activity assays; classification by catalytic function; thermal stability testing	Used in food processing aids and functional ingredient production
Short‐chain fatty acids (SCFAs)	Produced by colonic bacteria (Bacteroides, Firmicutes) via prebiotic fermentation (inulin, FOS)	Acetate, propionate, butyrate (~60:20:20 M ratio in colon)	Epithelial regeneration; NF‐κB suppression; barrier integrity; energy source for colonocytes; glucose/insulin modulation; weight management	GC/HPLC quantification of acetate:propionate:butyrate ratio; colonic pH measurement	Most extensively researched postbiotic class; applicable in metabolic‐health‐targeted functional foods
Bacteriocins	Ribosomally synthesized by LAB	Small peptides/proteins	Antimicrobial activity (membrane pore formation, cell wall assembly inhibition, enzyme/protein inhibition); alleviation of intestinal disease symptoms; metabolic regulation	Antimicrobial susceptibility testing; peptide sequencing; mode of action assays	Natural antimicrobial/biopreservative in food systems
Vitamins	Produced by various intestinal/fermentative bacteria	Heat‐sensitive micronutrient compounds	DNA replication, repair, and methylation support	Stability testing under heat exposure; quantification via HPLC	Limited by heat sensitivity; relevant for minimally processed or fortified foods
Neurotransmitters	*Bifidobacterium*, *Lactiplantibacillus plantarum*, *Lactobacillus brevis* , *Bacillus subtilis*	Serotonin, dopamine, norepinephrine, catecholamines, acetylcholine	Modulation of enteric neural signaling via the gut‐brain axis	Quantification via LC–MS/HPLC; gut‐brain axis bioassays	Emerging interest for gut‐brain targeted functional foods
Extracellular vesicles (EVs)	Released by commensal bacteria ( *Escherichia coli* , *Akkermansia muciniphila* )	Spherical lipid bilayer‐enclosed particles containing proteins, DNA, RNA, glycolipids, polysaccharides, enzymes, toxins	Microbial competition; pathogenesis modulation; immunomodulation; mucosal barrier penetration; systemic homeostasis; sepsis risk reduction	Vesicle isolation (ultracentrifugation); cargo profiling (omics); particle size/morphology characterization	Emerging delivery vehicle for bioactive compounds in functional foods

### 4.1 Inanimate Cells (Paraprobiotics)

As previously stated, the conventional definition of probiotics stipulates that the bacteria must be viable (Hill et al. 2014). However, bacterial viability is not strictly necessary to achieve all probiotic related effects, as many mechanisms and clinical benefits are not directly dependent on the survival of the bacteria. This phenomenon has been recently demonstrated by various studies comparing viable and inactivated microorganisms, as well as their distinct microbial fractions (Cuevas‐González et al. 2020). Consequently, the term “paraprobiotic” has been introduced to denote the “use of inactivated (non‐viable) microbial cells with the aim of conferring a health benefit to the consumer”. In the literature, paraprobiotics have been previously referred to as “inactivated probiotics” or “ghost probiotics” (de Almada et al. 2016; Divsalar et al. 2025). In a panel convened by ISAPP, inactivated microorganisms were also incorporated into the postbiotic classification, which has since been established as an umbrella term for these components (Salminen et al. 2021).

Findings derived from both in vitro and in vivo analyses have demonstrated that although paraprobiotics consist of cells lacking the capacity to proliferate and colonize the intestinal microbiota, their beneficial effects are comparable to those exhibited by viable probiotics. This phenomenon, which presents seemingly contradictory results, has been termed the “probiotic paradox” (Adams 2010; Mudaliar et al. 2024). While further research is warranted to fully elucidate the underlying mechanisms of action for paraprobiotics, existing studies have provided evidence supporting their health promoting effects, such as the enhancement of the immune system and the production of beneficial microbial metabolites, including peptides and SCFAs (Mudaliar et al. 2024).

### 4.2 Cell‐Free Supernatants and Suspensions

Cell‐free supernatants (CFS) represent a broad category of biomolecules and active metabolites with varying molecular weights, ranging from low to high, typically secreted by LAB and yeasts. These supernatants facilitate the maintenance of host homeostasis and include components such as organic acids, diacetyl, carbon dioxide, and bacteriocin‐like inhibitory substances (BLIS) (Gurunathan et al. 2024; Siedler et al. 2019). The supernatant comprises both the residual nutrients from the growth medium and the secondary metabolites resulting from microbial proliferation. However, the quantity, biochemical profile, and overall efficacy of these bioactive compounds within the CFS are highly variable. This variability arises from several critical factors, including the specific microbial strain utilized, the composition of the culture media, the duration and conditions of the fermentation process, and the downstream purification or extraction methods employed for metabolite recovery (Garg et al. 2026; Gurunathan et al. 2024). Despite this inherent variability, properly optimized CFS exhibit potent antibacterial, anti‐biofilm, anti‐inflammatory, antioxidant, and anti‐cancer activities. Furthermore, they demonstrate therapeutic potential in alleviating metabolic disorders and are utilized in the clinical management of diarrhea (Gurunathan et al. 2024; Lee et al. 2022; Wei et al. 2024).

### 4.3 Cell Wall Fragments

The bacterial cell wall comprises various components, including peptidoglycan, teichoic acids, and lipoteichoic acids. Peptidoglycan is generally composed of glycan chains of variable lengths, which are cross‐linked via oligopeptide bridges to provide the cells with the capacity to respond to environmental stress. Throughout the bacterial life cycle, peptidoglycans undergo continuous synthesis, remodeling, and repair processes to support cell division and expansion. Simultaneously with the constant synthesis of new peptidoglycan, older fragments are released into the surrounding environment, exerting beneficial effects on host health, such as immune system regulation and anti‐inflammatory activity. Peptidoglycans primarily manifest their anti‐inflammatory effects by modulating the expression of pro‐inflammatory cytokines. Teichoic acids, lipoteichoic acids, and other related immunogenic constituents of the bacterial cell wall can trigger the initiation of an immune response within the host's immune system. Furthermore, teichoic acids are essential for the pathophysiology and the development of antibiotic resistance (Gurunathan et al. 2024; van der Es et al. 2017; Wei et al. 2024). However, the immunomodulatory role of these cell wall components exhibits a dual nature, capable of either dampening or amplifying inflammatory responses depending on their specific structural features, concentration, and the host's microenvironment (Ding et al. 2026). While a moderate stimulation of pro‐inflammatory cytokines can be highly beneficial for maintaining immunological balance and increasing resistance to infections (Adams 2010), the inflammatory potential of these fragments poses significant safety concerns (Adams 2010; Liang and Xing 2023). High concentrations or excessive exposure to immunogenic fragments like lipoteichoic acid and peptidoglycan can provoke immune overstimulation, leading to an exaggerated inflammatory response that may negatively impact gut barrier function and exacerbate intestinal tissue damage (Ding et al. 2026; Liang and Xing 2023). For instance, certain peptidoglycan fragments have been shown to aggravate conditions like non‐alcoholic steatohepatitis by overactivating inflammatory signaling pathways (Ding et al. 2026). Because these components directly activate toll‐like receptors and other pattern recognition receptors (Ding et al. 2026; López‐Almada et al. 2024), uncontrolled immune activation is a particular risk for susceptible populations, such as neonates, the elderly, immunocompromised individuals, or patients with pre‐existing inflammatory disorders (Ding et al. 2026; Piqué et al. 2019). Consequently, despite their therapeutic promise, a careful safety assessment including rigorous screening of bacterial strains and strict optimization of dosage, formulation, and delivery methods is absolutely essential to harness their immunomodulatory benefits without triggering detrimental inflammation (Adams 2010; Ding et al. 2026; Liang and Xing 2023).

### 4.4 Exopolysaccharides

Exopolysaccharides (EPS) are high molecular weight extracellular carbohydrate polymers secreted by microorganisms. These macromolecules possess defensive capabilities against phages, phagocytic cells, and toxic substances. They also influence the immune system, physiological processes, lipid metabolism, and pathogenic colonization within the host. Centrifugation constitutes the initial stage of a multi phase method used for the recovery of EPS, followed by the stages of protein removal (deproteinization), filtration, dialysis, and lyophilization. EPS are essential compounds for cell adhesion and defense. The structural diversity of EPS produced by LAB enables these polymers to exhibit a range of bioactivities, including immunomodulatory, anti‐tumor, anti‐mutagenic, anti‐inflammatory, antioxidant, anti‐hypertensive, antiviral, antibacterial, anti‐gastrointestinal, and cholesterol lowering activities (Abdalla et al. 2021; Angelin and Kavitha 2020; Caggianiello et al. 2016; Gurunathan et al. 2024; Wei et al. 2024). To date, these diverse functionalities have been extensively demonstrated primarily through in vitro assays and in vivo animal models (Kaya et al. 2026; Rafique et al. 2023). Furthermore, EPS have been successfully utilized in various food systems such as dairy, bakery, and meat products where they demonstrate both technological improvements and bioactive health benefits (Homayouni‐Rad et al. 2025). However, comprehensive clinical validation of these specific therapeutic effects in human systems remains relatively limited (Kaya et al. 2026). While the existing data is highly promising, more rigorous and large scale clinical trials are required to fully establish and confirm their efficacy in human populations (Liang and Xing 2023; Rafique et al. 2023).

### 4.5 Enzymes

Enzymes represent a class of proteins responsible for catalyzing biological reactions and are broadly categorized into six principal groups according to their functional activities: hydrolases, oxidoreductases, isomerases, transferases, ligases, and lyases (Kumar et al. 2017). A limited number of bacterial strains, specifically *Bacillus licheniformis* and *Bacillus subtilis* , as well as several fungal strains, notably *Aspergillus oryzae* and *Aspergillus niger*, serve as primary sources for enzymes utilized in various physiological, metabolic, and regulatory processes (Contesini et al. 2018; Gurunathan et al. 2024). When utilized as postbiotics within a food matrix, these microbial enzymes exhibit complex behaviors that can serve as technological aids but also present stability challenges. Functionally, postbiotic enzymes such as proteases, lipases, esterases, and amylases actively break down matrix macromolecules, which significantly improves the texture, tenderness, aroma, and flavor profiles of products like meat, dairy, and fermented foods (Akter et al. 2026; Homayouni‐Rad et al. 2025). Furthermore, specific postbiotic enzymes like phytases can degrade anti‐nutritional factors in cereal matrices, thereby increasing the bioavailability of essential minerals (Karabacak Aydin et al. 2026; Mishra et al. 2024). However, the efficacy of these enzymes is highly susceptible to the internal conditions of the food matrix. The bioactivity of postbiotic enzymes can be compromised or deactivated by interactions with other food components or by endogenous food borne proteolytic enzymes present in the matrix (Aggarwal et al. 2022; Divsalar et al. 2025). Conversely, if not properly controlled, an excessive concentration of active postbiotic lipolytic and proteolytic enzymes can over hydrolyze the food components, leading to accelerated proteolysis and undesirable sensory alterations, such as off‐flavors or degraded textures. Therefore, balancing their concentration or applying targeted inactivation techniques is often required to prevent excessive degradation and optimize their application in food systems (Divsalar et al. 2025; Sharafi et al. 2023).

### 4.6 Short‐Chain Fatty Acids

Short‐chain fatty acids (SCFAs) represent a crucial class of compounds produced by intestinal bacteria, like *Bacteroides* and *Firmicutes*. Prebiotics like inulin and fructooligosaccharides are fermented by these microbes to produce SCFAs, primarily acetate, propionate, and butyrate. These fatty acids are typically found in the colon in a molar ratio of approximately 60:20:20 and facilitate the regeneration of the intestinal epithelium. Furthermore, they prevent the activation of nuclear factor‐kappa B (NF‐κB) by suppressing the production of pro‐inflammatory cytokines. As the most extensively researched postbiotics, SCFAs exert numerous beneficial effects on health. In addition to improving colonic function and lowering luminal pH, they stimulate the proliferation of colonic epithelial cells and enhance blood flow. SCFAs also maintain the integrity of the intestinal barrier, serve as a vital energy source for colonocytes, and modulate the immune system, glucose metabolism, and insulin sensitivity. Consequently, they play a significant role in energy metabolism, nutrition, and weight management (Fattahi et al. 2020; Gurunathan et al. 2024; Kim et al. 2018; Wei et al. 2024; Zhong et al. 2024; Żółkiewicz et al. 2020). When incorporated into food formulations, SCFAs function as effective natural bio‐preservatives by reducing the pH of the food matrix, which inhibits the growth of spoilage microorganisms and foodborne pathogens and extends product shelf life (Akter et al. 2026; Garg et al. 2026). However, their direct application is often limited by their distinct sensory properties; at functional concentrations, SCFAs can impart strong acidic, sour, or vinegary off‐flavors that may negatively impact consumer acceptance (Akter et al. 2026; Issazadeh and Hatami 2025). To mask these undesirable sensory attributes and prevent rapid dilution and premature gastrointestinal degradation, advanced postbiotic delivery systems are increasingly utilized. Encapsulation techniques such as microencapsulation, nanoemulsions, and hydrogels are widely employed to enhance SCFA stability during food processing and storage (Karabacak Aydin et al. 2026). Furthermore, to maximize their health benefits in the host, colon targeted oral delivery systems, often utilizing pH dependent polymers or engineered nanoparticles, are designed to protect SCFAs through the acidic environment of the upper digestive tract and ensure their controlled release directly into the lower intestine (Abbasi et al. 2022; Karabacak Aydin et al. 2026).

### 4.7 Bacteriocins

Bacteriocins constitute a class of small, ribosomally synthesized peptides or proteins that are produced by lactic acid bacteria (LAB). These molecules inhibit the growth of pathogens within the gastrointestinal tract by creating pores in cell membranes, preventing the proper assembly of cell walls, and inhibiting essential enzyme and protein functions (Gurunathan et al. 2024; Soltani et al. 2021; Wei et al. 2024). Beyond their antimicrobial activity, the physiological functions of bacteriocins include the alleviation of intestinal disease symptoms and the contribution to metabolic regulation (Patra et al. 2022; Wei et al. 2024). Among these antimicrobial peptides, nisin like compounds (particularly nisin) are extensively utilized in food biopreservation (Aggarwal et al. 2022; Aguilar‐Toalá et al. 2018). Nisin is globally approved as a safe food additive and is highly effective at inhibiting dangerous foodborne pathogens, such as *Listeria monocytogenes* and *Clostridium botulinum* , across various dairy, bakery, and meat products (Aguilar‐Toalá et al. 2018; Garg et al. 2026). However, the successful commercial application of bacteriocins in food systems is heavily matrix dependent. Their antimicrobial activity can be significantly reduced when directly applied to complex foods due to interactions with specific food components. For example, nisin can be trapped by lipids in fat‐rich meat matrices, which drastically decreases its efficacy (Homayouni‐Rad et al. 2025). Furthermore, bacteriocins are highly susceptible to degradation by endogenous proteolytic enzymes present in the food matrix (Divsalar et al. 2025). To overcome these matrix dependent limitations and maintain their functional stability, bacteriocins are increasingly incorporated into advanced delivery systems, such as nano‐encapsulation, or immobilized within active packaging films, which protect the peptides from enzymatic degradation and undesired matrix interactions (Cheruvari and Kammara 2025; Divsalar et al. 2025).

### 4.8 Vitamins

Vitamins are heat sensitive components essential for the body to execute a range of physiological processes (Gurunathan et al. 2024; LeBlanc et al. 2013). Because many of these essential nutrients, particularly B group vitamins, are highly thermosensitive, they are frequently degraded or lost during conventional thermal food processing and milling operations (Nataraj et al. 2020; Suthar et al. 2025). To compensate for these nutritional losses, the use of vitamin producing microorganisms has emerged as a highly effective postbiotic application in the food industry (Gurunathan et al. 2024; Mishra et al. 2024; Nataraj et al. 2020). These beneficial microbes are capable of the synthesis of essential vitamins, including riboflavin, folate, cobalamin, and vitamin K, directly within the food matrix (Calvanese et al. 2025; Garg et al. 2026; Gurunathan et al. 2024). Utilizing these postbiotic metabolites for food bio‐fortification serves as a natural, cost effective, and sustainable alternative to the addition of chemically synthesized pseudo vitamins (Calvanese et al. 2025; Nataraj et al. 2020). This approach has been successfully implemented to enrich a variety of functional foods, including fermented milks, yogurts, cheeses, and cereal based products (Gurunathan et al. 2024; Suthar et al. 2025). Ultimately, integrating these microbial vitamins not only restores the nutrients lost during manufacturing but also significantly enhances the overall nutritional profile, bioavailability, and health promoting value of the final food products without causing adverse side effects (Gurunathan et al. 2024; Mishra et al. 2024; Nataraj et al. 2020).

### 4.9 Neurotransmitters

Neurotransmitters such as dopamine, serotonin, catecholamines, acetylcholine, and norepinephrine are synthesized by various intestinal bacteria, including *Bifidobacterium*, *Lactiplantibacillus plantarum*, *Bacillus subtilis* , and *Lactobacillus brevis* . These neurotransmitters play a critical role in brain function by modulating enteric neural signaling via the gut‐brain axis (Aggarwal et al. 2022; Gurunathan et al. 2024). In the food industry, the application of these microbial neuroactive compounds, particularly gamma aminobutyric acid (GABA) and serotonin precursors, has emerged as a highly successful strategy for developing advanced functional foods (Calvanese et al. 2025; Icer et al. 2024). Because LAB naturally synthesize high levels of neurotransmitters like GABA via the glutamate decarboxylase pathway, specific strains such as *Levilactobacillus brevis* and *Lactiplantibacillus plantarum* are extensively utilized as starter cultures to naturally bio‐enrich food matrices during fermentation. This targeted microbial activity has led to the widespread commercialization of neurotransmitter‐enriched products, including dairy, cereal, and bakery goods, legumes, and functional beverages. Furthermore, isolated postbiotic GABA is directly utilized as a safe and stable food additive in everyday products such as chocolates, biscuits, and potato snacks. Utilizing these microbial neurotransmitters in food formulations not only serves as a cost‐effective, non‐pharmacological delivery system to confer targeted neuropsychiatric and physiological benefits, but the associated fermentation processes also simultaneously improve the organoleptic properties of the final food products (Icer et al. 2024).

### 4.10 Extracellular Vesicles

Extracellular vesicles (EVs) are spherical, lipid bilayer enclosed particles released into the environment by commensal bacteria such as *Escherichia coli* and *Akkermansia muciniphila* . These vesicles contain a diverse array of cargo, including DNA, RNA, proteins, enzymes, polysaccharides, glycolipids, and toxins, and they play pivotal roles in microbial competition, pathogenesis, and immunomodulation. Furthermore, EVs can rapidly penetrate the mucosal barrier and interact directly with the host. Through these mechanisms, they contribute to maintaining systemic homeostasis and reducing the risk of sepsis (Badi et al. 2017; Chelakkot et al. 2018; Gurunathan et al. 2024). However, the clinical and industrial application of EVs requires careful consideration of their safety profile and comprehensive characterization. A major safety concern arises from the potential presence of pore forming toxins and lipopolysaccharides (LPS) in EVs derived from Gram‐negative bacteria, which can induce strong immune responses, reactogenicity, and excessive inflammation in vulnerable populations (Karabacak Aydin et al. 2026; Xie et al. 2024). Furthermore, the safety and efficacy of EVs are highly species and strain specific. For instance, the LPS structure varies among bacteria, and LPS derived from *A. muciniphila* provokes a significantly weaker inflammatory response compared to that from *E. coli* (Xie et al. 2024). Another significant challenge is the inherent variability of EV cargo. The EV population is extremely heterogeneous in its density, cargo composition, and size, which fluctuates based on the biogenesis route, the genetic background of the specific producing strain, and external growth conditions. Finally, the precise characterization and quantification of specific EVs remain technically difficult. This is due to their nanoscale size, the complexity of biological mixtures, the lack of specific EV recognizing antibodies, and the requirement for sophisticated, time consuming isolation techniques such as ultracentrifugation and size exclusion chromatography (Ding et al. 2026; Xie et al. 2024).

## 5 Identification and Enumeration Methods for Postbiotics

**FIGURE 5:**
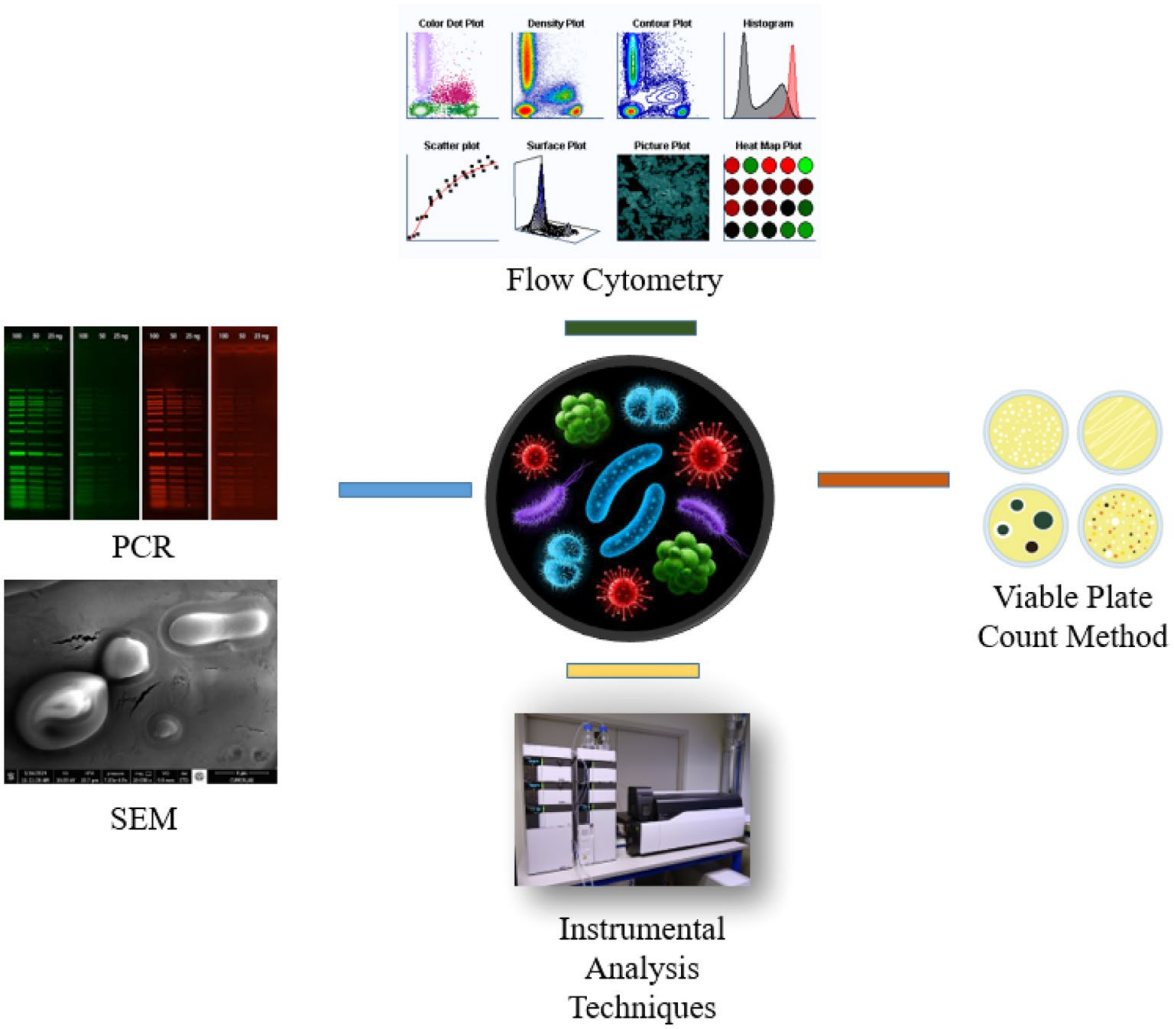
Identification and enumeration methods for postbiotics.

In the identification of postbiotics, the selection of the appropriate instrumental technique is determined by the analytical objectives and the specific type of characterization required. The traditional plate count technique is utilized in food samples to evaluate cell viability for microbial identification. This method serves as a quality control measure to verify the presence of inactivated cells resulting from various inactivation processes applied to probiotics (Barros et al. 2020; Wilkinson 2018). However, it is time consuming and significantly underestimates cellular populations by failing to detect viable but not culturable (VBNC) or dead cells. To overcome these limitations, flow cytometry has emerged as a superior, rapid alternative that accurately differentiates among live, damaged, and dead cells (Boyte et al. 2023). Flow cytometry is recognized as a relatively novel approach used to evaluate alterations in bacterial cell morphology and metabolism induced by different processing treatments, storage durations, and simulated gastrointestinal conditions. In addition to being the superior method for detecting inactivated cells and assisting in the selection of optimal processing conditions, flow cytometry also contributes to a more comprehensive understanding of the mechanisms of action through which inactivated cells exert their health promoting effects (Barros et al. 2020; Wilkinson 2018). The assessment of cell membrane integrity and the detection of metabolic activity can be effectively performed using flow cytometry (Barros et al. 2020; Vinderola et al. 2019). Conversely, while flow cytometry generally lacks strain specificity, culture independent methods such as real time quantitative PCR (qPCR), reverse transcriptase PCR (RT‐PCR), propidium monoazide PCR (PMA‐PCR) generally oriented toward the detection of nucleic acids. These methods offer high precision and strain level differentiation, albeit requiring extensive primer optimization. Also, to identify postbiotic metabolites, matrix assisted laser desorption/ionization mass spectrometry (MALDI‐TOF) is utilized, while HPLC and proton nuclear magnetic resonance spectroscopy (H‐NMR) are employed to identify and characterize polysaccharide‐glycopeptide complexes. Scanning electron microscopy (SEM) can also be utilized for morphological examination. Furthermore, chromatography coupled with tandem mass spectrometry (LC–MS/MS) and direct infusion fourier transform ion cyclotron resonance mass spectrometry (FT‐ICR‐MS) is used to identify and characterize metabolites such as fatty acids, glycerolipids, oligosaccharides, sphingolipids, and purines within biological samples. On the other hand, techniques such as ultra performance liquid chromatography (UPLC), which provide high efficiency and resolution, low solvent consumption, and high accuracy and sensitivity, are also extensively preferred (Aguilar‐Toalá et al. 2018; Antunes et al. 2011; Dong and Guillarme 2013; Kim et al. 2011). Figure 5 presents the methods and instrumentation employed for the analysis and characterization of postbiotics. The figure demonstrates that the analysis of microorganisms possesses a multi‐layered structure, indicating that viability assessment, genetic verification, morphological examination, and chemical component analyses serve as complementary methods.

## 6 Benefits and Advantages of Postbiotics

Postbiotics are considered advantageous and beneficial, not only because they do not give rise to adverse effects, but also due to their positive health promoting properties and their wide range of potential applications in food technology (Aggarwal et al. 2022; Hernández‐Granados and Franco‐Robles 2020; Mokashe et al. 2026). Numerous studies in the literature have focused on the antioxidant, antidiabetic, antibacterial, and antimicrobial effects, microbial inactivation mechanisms, safety profiles, and functional potentials of postbiotics derived in various forms from different bacterial species (Amobonye et al. 2025; Cuevas‐González et al. 2020; Gurunathan et al. 2024). Furthermore, the metabolite profiles, immunomodulatory effects, biological and physicochemical properties, viability and functional attributes, mechanisms of action, and active components of postbiotic preparations have been examined (Aguilar‐Toalá et al. 2018; Amobonye et al. 2025; Baheti et al. 2026). Additionally, studies investigating the effects of utilizing next generation technologies, developed particularly in recent years and applied either alone or in combination, on the structural, functional, and biological characteristics of postbiotics are also present in the literature (Balthazar et al. 2022; Moghadam et al. 2026; Thirumdas and Mudgil 2025). The objectives and key findings of the research conducted within this scope are summarized in Table 3.

**TABLE 3:** Main characteristics of different types of postbiotics.

Source microorganism/postbiotic component	Objective	Key outcomes	References
*Lactiplantibacillus plantarum* O7S1 supernatant and EPS	Investigating the in vitro antioxidant, antidiabetic, and antibacterial properties of postbiotics produced during the fermentation process	The hypoglycemic, antioxidant, and antibacterial properties displayed by both the supernatant and EPS point to their promise as alternative candidates for use in functional foods and pharmaceuticals targeting the prevention of oxidative stress, diabetes, and pathogenic bacterial infections	Aliouche et al. (2024)
*Lactiplantibacillus plantarum* DSM 9843, *Lacticaseibacillus rhamnosus* GG, *Bifidobacterium longum* 35,624, *Bifidobacterium breve* DSM 16604	Optimizing the cofermentation conditions of *Lactobacillus* and *Bifidobacterium* strains to maximize postbiotic yield and biofunctional properties	Employing equal weighting in the multi‐response optimization approach, the optimal processing conditions were determined to be a 2.5% inoculum size, a fermentation duration of 48 h, and mild heat treatment, yielding a composite desirability value of 0.9919. Under these optimized parameters, postbiotic yield attained 70.70 mg/mL, antioxidant activity reached 80.19%, α‐glucosidase inhibition was recorded at 72.50%, α‐amylase inhibition at 84.38%, and EPS concentration at 648.01 μg/mL. Among the tested variables, inoculum size and heat treatment emerged as the most influential factors, followed by fermentation time. The application of mild heat treatment enhanced postbiotic yield, antioxidant activity, α‐amylase inhibition, EPS concentration, and the concentrations of B‐group vitamins (B_2_, B_3_, B_6_, and B_9_), while α‐glucosidase inhibition showed a marginal increase under non‐heat‐treated conditions. Furthermore, the optimized postbiotics demonstrated broad‐spectrum antimicrobial activity against both Gram‐negative and Gram‐positive bacteria, with enhanced efficacy observed in the mild heat‐treated samples	Asefa et al. (2026)
*Lacticaseibacillus rhamnosus* ATCC 53103	Evaluating the microbial inactivation mechanism using *Lacticaseibacillus rhamnosus* ATCC 53103 as a model organism under pressure or thermal treatments	The double staining technique using the flow cytometry propidium iodide and fluorochromes carboxyfluorescein diacetate revealed that heat‐killed cells exhibited distinct fluorescence behaviors depending on the temperature regime applied. While cells killed at 60°C remained unstained, those heat‐treated at 75°C formed a single population labeled with propidium iodide. These results indicate that heat‐induced cell death may take place regardless of whether membrane degradation occurs. By contrast, hydrostatic pressures exceeding 400 MPa inactivated *L. rhamnosus* ATCC 53103 through a mechanistically distinct pathway, with high‐pressure‐induced cell death found to arise predominantly from irreversible damage to membrane‐bound transport systems	Ananta and Knorr (2009)
Postbiotics derived from LAB isolated from fermented tarhana (CFS and heat‐killed cell fragments)	Evaluating the antimicrobial activity of postbiotics against *Bacillus cereus* , *Listeria monocytogenes* , *Staphylococcus aureus* , *E. coli* , and *Salmonella typhimurium*	In total, 150 postbiotic samples were derived from 75 LAB isolates. It was determined that 41 of these inhibited *S. aureus* and 18 inhibited *L. monocytogenes* , with IC50 values ranging from 8.52 to 9.20 mg/mL, and that there was no significant difference between the CFS and heat‐killed cell forms of the postbiotics in terms of activity or structure	Aydin et al. (2025)
*Lactobacillus acidophilus* and *Lactobacillus helveticus*	Investigating the effects of high‐intensity ultrasound (at 20%, 30%, and 40% amplitudes for 3 min) on the growth and fermentation profiles of *Lactobacillus acidophilus* and *Lactobacillus helveticus*	For both probiotic strains, galactosidase activity increased at 20% and 30% amplitudes compared to the control, whereas it decreased at 40% amplitude. Notably, the lowest proteolysis values were observed at 30% amplitude. While acetic, citric, and lactic acids were the predominant organic acids, formic acid was not detected in either samples	Bolívar‐Jacobo et al. (2023)
*Lactiplantibacillus plantarum* and *Lactobacillus gasseri*	Investigating the effects of thermal and sonication inactivation methods on probiotics and to determine a suitable inactivation method for the production of postbiotics from *Lactiplantibacillus plantarum* and *Lactobacillus gasseri*	The results revealed that each strain exhibited varying resistance to the inactivation methods. Heat‐treated *Lactobacillus gasseri* (at 75°C and 90°C for 15 and 30 min) lost its growth ability, whereas samples subjected to sonication (at 50 and 100 W for 5 and 10 min) retained their viability. In the case of *Lactiplantibacillus plantarum*, it was determined that the growth capability in the culture medium following sonication was lower than that of the heat‐treated samples. Flow cytometric analysis revealed that membrane integrity in both probiotic strains was disrupted by each of the inactivation methods applied; nevertheless the most severe membrane damage in *Lactobacillus gasseri* cells was observed following severe heat treatment (90°C for 30 min). The morphological characteristics of both strains exhibited significant susceptibility to the inactivation methods applied. Following the inactivation treatments, significant increases were observed in damaged cells, cell roughness, lysed cells, cell adhesion, cell disruption, and cell twisting	Gholian et al. (2024)
Postbiotic ATA‐LTW 35	Investigating the antimicrobial effects of liposomal postbiotics formulated as a gel	It was determined that the formulated postbiotics exhibited antimicrobial activity comparable to that of free postbiotics; thus, liposomal gel formulations enhance the antimicrobial activity of postbiotics while providing practical benefits in terms of application	Gökçe and Aslan (2024)
*L. plantarum* IZITR_24 and *L. paraplantarum* IZITR_13 lyophilized CFS	Investigating the anti‐staphylococcal efficacy of two *Lactiplantibacillus* strains isolated from traditional Bulgarian pickled vegetables (turshiya): *L. plantarum* IZITR_24 and *L. paraplantarum* IZITR_13	Both strains exhibited pronounced, dose‐dependent inhibition of *S. aureus* growth as well as biofilm formation. These findings were further corroborated by microscopic analyses (SEM/CLSM), which confirmed substantial disruption of the biofilm structure along with permeabilization of the cell membrane	Ilieva et al. (2026)
Postbiotic components of *Limosilactobacillus reuteri* and *Lacticaseibacillus rhamnosus*	Investigating the safety and antibacterial properties of the relevant postbiotic components (cell free extracts) for their potential application in food products	While total flavonoid content (1971.79 ± 20 mg Qu/g), total SCFAs (23 μg/g), sugar content, and the antioxidative enzymes CAT and SOD were detected at higher levels in *Lacticaseibacillus rhamnosus* postbiotics, GSH‐px levels and riboflavin were found to be higher in the postbiotics of *Limosilactobacillus reuteri*. No significant difference was recorded in the total phenolic (2501 and 2518 mg GAE/L) and protein contents (305.58 and 296.23 μg/g) of the postbiotics. Both freeze‐dried postbiotic samples were more effective against Gram‐positive pathogens compared to Gram‐negative pathogens, whereas their antibacterial effects increased when the two postbiotics were used in combination. Neither postbiotic sample exhibited hemolytic activity against human erythrocyte cells, and both showed low cytotoxicity in MRC‐5 and IPEC‐J2 cell lines at the highest concentrations used (150 mg/mL)	Jalali, Mojgani, Haghighat, et al. (2024), Jalali, Mojgani, Sanjabi, et al. (2024)
*Bifidobacterium animalis* subsp. *lactis* BB‐12	Evaluating the viability and functional properties of postbiotics derived from *Bifidobacterium animalis* subsp. *lactis* BB‐12 following thermal and ultrasound processing	Complete microbial inactivation was observed across all heat‐treatment conditions; in contrast, total loss of culturability was achieved in only 11 of the ultrasound conditions, suggesting that microorganisms exhibit greater resistance at lower intensities or shorter treatment durations. Postbiotics derived from heat treatment demonstrated markedly stronger probiotic‐enhancing properties, with growth of *L. casei* increasing up to 2.9‐fold and that of *Bifidobacterium* spp. up to 37.7‐fold relative to controls, in addition to superior antimicrobial activity, particularly against *Enterococcus faecalis* . Notably, both processing approaches led to significant increases in short‐chain fatty acid levels	Marsak et al. (2025)
*Streptococcus lutetiensis*	Investigating the functional potential, probiotic properties, postbiotic preparations, metabolite profile, and immunomodulatory effects of *Streptococcus lutetiensis*	In vitro investigations employing THP‐1 macrophages demonstrated that postbiotics obtained from *Streptococcus lutetiensis* modulated cytokine secretion profiles in a fashion comparable to that of live probiotics. Of the 27 cytokines examined, 15 were found to be secreted in response to both postbiotic and probiotic treatments. Notably, postbiotics elicited elevated levels of IL‐8 and IL‐5, along with increased concentrations of G‐CSF, IL‐10, and MIP‐1b. Furthermore, metabolite profiling conducted via GC–MS and HPLC identified a range of compounds including fatty acids, organic acids, peptides, alcohols, aldehydes, ketones and esters that contribute to the observed antimicrobial and antioxidant activities	Mehta et al. (2025)
*Lactiplantibacillus plantarum* UTNGt2, *Lactococcus lactis* UTNGt28, *Weissella cibaria* UTNGt21O cell‐free supernatant and EPS	Investigating the biological and physicochemical properties of postbiotic‐based extracts	Among the formulations tested, FU6 (CFS UTNGt28: EPS UTNGt2) and FU13 (CFS UTNGt21O) emerged as the most potent. Damage to the bacterial membrane and cell wall, exhibiting both dose‐ and time‐dependent characteristics, was confirmed through and scanning electron microscopy (SEM) and transmission electron microscopy (TEM). Notably, FU6 displayed superior antioxidant capacity and no hemolytic activity, while both FU6 and FU13 elicited cell‐line‐specific responses when tested against HT‐29 (intestinal mucus‐producing) and HEK293 (human kidney) cells. Furthermore, characteristic absorption bands corresponding to lipids, proteins, nucleic acids, and carbohydrates were detected via attenuated total reflectance‐Fourier transform infrared (ATR‐FTIR) spectroscopy, while proton nuclear magnetic resonance analysis allowed for the identification of principal amino acids, monosaccharides, and metabolites such as acetate and lactate present in the extracts	Molina et al. (2025)
*Lactobacillus acidophilus* LA‐5 heat‐killed and ultrasound treated cells	Evaluating the viability and functional properties of *Lactobacillus acidophilus* LA‐5 following heat treatment and ultrasound application	While all heat treatment temperatures completely inactivated the probiotics, 9 ultrasound conditions were effective for inactivation. Heat‐treated postbiotics markedly promoted the growth of *L. casei* 431, yielding up to a 10.8‐fold increase relative to the control, while ultrasound‐treated postbiotics exerted only a negligible effect. The most pronounced antimicrobial activity was recorded in heat‐treated postbiotics, resulting in population reductions of 14.6% and 8.4% for *E. coli* and *Enterococcus faecalis* , respectively; in contrast, these rates were recorded as 10% and 4% in ultrasound‐treated samples. An increase in the level of SCFAs was observed following both methods	Müldür et al. (2025)
*Bifidobacterium bifidum* BB‐12	Establishing systematic knowledge regarding the effect of spray drying on the viability of *B. bifidum* BB‐12	*B. bifidum* BB‐12 was significantly damaged by the drying process, exhibiting severe membrane damage, depolarization, and reduced microbial proliferation. Gum arabic, gelatin, and pectin were identified as the agents providing the most effective protection. In cells treated with these substances during spray drying, reduced membrane damage and enhanced stability were observed throughout the storage period	Salar‐Behzadi et al. (2013)
*Lactiplantibacillus plantarum* MIUG BL21	Investigating the effects of different inactivation methods, applied individually (thermal, ohmic heating, high pressure, and ultrasound) and in combination (ohmic heating, high pressure, and ultrasound combined with thermal heating), on the inactivation kinetics of *Lactiplantibacillus plantarum*	Cytocompatibility along with antiproliferative activity was observed in the samples, as evidenced by enhanced proliferation in murine fibroblast cells as well as in the human colorectal adenocarcinoma cell line. Nonetheless, further investigation is required to characterize and quantify the postbiotic compounds liberated through the different inactivation treatments employed	Stănciuc et al. (2024)
*Lactobacillus delbrueckii* subsp. *bulgaricus* and *Streptococcus salivarius* subsp. *thermophilus*	Investigation of the effect of thermal processing on bioactive peptide compounds	According to the findings, thermal inactivation contributed to a greater diversity of bioactive peptides, with lower‐molecular‐weight fractions being particularly affected. Notably, peptides generated through thermal processing with molecular weights below 3 kDa were reported to predominantly display potential bioactivity as inhibitors of angiotensin‐converting enzyme and dipeptidyl peptidase‐IV	Sulistiawati et al. (2024)
*Lacticaseibacillus paracasei* ET‐22 and *Bifidobacterium lactis* BL‐99	Evaluating the effect of thermal inactivation temperature on the antioxidant and anti‐inflammatory properties, and chemical composition of ET‐22 and BL‐99 postbiotics	Subjecting ET‐22 and BL‐99 to thermal treatment across a temperature range of 70°C to 121°C for a duration of 10 min resulted in effective microbial inactivation, accompanied by disruption of cellular architecture and subsequent release of intracellular contents. Notably, while temperatures below 100°C left the anti‐inflammatory and antioxidant activities of these postbiotics largely unaltered, exposure to 121°C led to a marked decline in the levels of several bioactive constituents, including 6‐hydroxyhexanoic acid, phenyllactic acid zomepirac, and flumethasone	Sun et al. (2023)
*Lactococcus lactis*	Investigating the functionality of two EPS‐producing *Lactococcus lactis* strains, which differ significantly in their techno‐functional properties, in concentrated acid milk gel suspensions in relation to the structural and macromolecular properties of the EPS	The strains produced EPS with distinct characteristics in terms of gel hardness, improvement in whey separation, particle size, and the creamy texture imparted to the product. Structural and molecular characterization of the isolated EPS revealed that the ropy variant, characterized by a larger hydrodynamic radius and a reduced degree of branching, conferred improved texture and stability in the low‐fat model fresh cheese	Surber et al. (2021)
*Bacillus amyloliquefaciens* J and *Lactiplantibacillus plantarum* SN4	Investigatig the antimicrobial activity, mechanism of action, and active components of a novel postbiotic recently developed from *Bacillus amyloliquefaciens* J and *Lactiplantibacillus plantarum* SN4	The postbiotic extract exhibited potent bactericidal activity against a broad spectrum of pathogens, including *E. coli* , *Staphylococcus aureus* , and *Salmonella typhimurium* . Among the antibacterial constituents detected in the extract, organic acids emerged as the dominant contributors, with lactic acid (378 ± 4.1 mg/g) and acetic acid (40.8 ± 2.2 mg/g) representing the two most abundant species. Furthermore, the extract demonstrated high stability under enzymatic degradation and thermal processing	Tong et al. (2025)
*Lactiplantibacillus plantarum* 299v, *Lacticaseibacillus casei Shirota* and *Bifidobacterium animalis* subsp. *lactis* BPL1	Analyzing the effect of inulin and chia mucilage on short‐chain fatty acid production and the antioxidant activity of the supernatant as a postbiotic in a whey‐based culture medium	*Lactiplantibacillus plantarum* 299v and *Lacticaseibacillus casei Shirota* were found to produce lactic and acetic acids, whereas *Bifidobacterium animalis* subsp. *lactis* BPL1 demonstrated the greatest short‐chain fatty acid output when cultured in medium supplemented with 2% inulin. It is worth noting that the antioxidant activity exhibited by postbiotics obtained from *Lactiplantibacillus plantarum* 299v was markedly improved through the addition of soluble fibers	Vera‐Santander et al. (2024)
Direct‐feed starter cultures of three probiotic strains, namely LCZ, V9, and P‐8	Investigating the metabolomics profile of a potential postbiotic produced by co‐fermentation using three probiotic strains	A markedly elevated content of several metabolite classes characterized the candidate postbiotic, encompassing amino acids and their derivatives, nucleotides and associated metabolites, aldehydes, ketones, fatty acids, glycerolipids, tryptamine, glycerophospholipids, esters, cholines, pigments, organic acids and heterocyclic compounds together with their derivatives. Among these, amino acids and organic acids stood out as the metabolites most clearly differentiating the postbiotic from the comparator samples	Wu et al. (2024)
*Bifidobacterium animalis* subsp. *lactis* BB‐12	Producing postbiotics from *Bifidobacterium animalis* subsp. *lactis* BB‐12 using ohmic heating via response surface methodology based on flow cytometry and spectrophotometric analyses	Lactic and acetic acids were identified as the predominant metabolites characterized in both probiotic and postbiotic samples. The optimum ohmic heating conditions were established as an electric field of 8 V/cm, a heating temperature of 88°C, a bacterial population of 8 log CFU/mL, and a heating duration of 3 min. Under these optimum conditions, the values for cell surface hydrophobicity, cell auto‐aggregation, co‐aggregation, and membrane integrity were recorded as 44.82%, 38.05%, 40.79%, and 94.82%, respectively	Yolmeh et al. (2024)
*Bifidobacterium animalis* subsp. *lactis* BB‐12	Obtaining postbiotics from *Bifidobacterium animalis* subsp. *lactis* BB‐12 exhibiting antimicrobial activity against *Salmonella enterica*	The major metabolites in *Bifidobacterium lactis* were identified as glycerol (37.6 μmol/g), ethanol (22.6 μmol/g), and lactate (9.8 μmol/g), whereas in the postbiotics, they were glycerol (47.8 μmol/g), acetate (34.0 μmol/g), and lactate (24.6 μmol/g). All anti‐ *Salmonella enterica* properties of the postbiotics obtained under optimal ohmic heating conditions were found to be higher than those of *Bifidobacterium lactis* (the untreated sample). The optimal ohmic heating conditions to obtain a postbiotic effective against *Salmonella enterica* were determined as an electric field of 8.7 V/cm, an ohmic heating temperature of 88°C, a cell concentration of 8.7 log CFU/mL, and an ohmic heating duration of 3.6 min	Yolmeh et al. (2025)
*Lactobacillus casei* subsp. *casei* SY13 and *Lactobacillus delbrueckii* subsp. *bulgaricus* LJJ	Evaluating the antioxidant activity of intact cells and cell‐free extracts of *Lactobacillus casei* subsp. *casei* SY13 and *Lactobacillus delbrueckii* subsp. *bulgaricus* LJJ isolated from traditional yogurt	Both *Lactobacillus* strains exhibited good antioxidant capacity, inhibiting linoleic acid peroxidation by 62.95% and 66.16% respectively. The antioxidant activity of intact cells was observed to be superior to that of cell‐free extracts	Shuwen (2011)
*L. plantarum* strains (RG11, RG14, RI11, RS5, TL1, and UL4)	The effects of different combinations of carbon (glucose, lactose, sucrose, and dextrose) and nitrogen (X‐SEED Kat, X‐SEED Peptone, X‐SEED Nucleo Advanced, Nucel875 MG, FM888, and FM902) sources on the growth of six *L. plantarum* strains (RG11, RG14, RI11, RS5, TL1, and UL4) and the functional properties of their respective postbiotic metabolites (bacteriocin inhibitory activity, antioxidant activity, and lactic acid concentration)	The functional characteristics of the postbiotic metabolites, as influenced by varying combinations of carbon and nitrogen sources, were found to be strain‐specific. Among the strains tested, UL4 displayed the greatest bacteriocin inhibitory activity when cultured with dextrose and the FM888 nitrogen source, while RI11 achieved the highest lactic acid concentration under dextrose and Nucel875 supplementation. The postbiotic metabolites of strains RS5 and TL1 exhibited maximal reducing power in MRS medium, whereas RG14 demonstrated the highest hydroxyl radical scavenging activity when sucrose was combined with X‐Seed KAT	Zheng et al. (2024)

## 7 Potential Applications of Postbiotics

The utilization of postbiotics in food systems serves a dual purpose, enhancing the nutritional value and shelf life of food products while simultaneously providing positive effects on human health. Postbiotics can improve the functional, nutritional, and sensory characteristics of foods. They generally exhibit stability across high temperatures and a wide pH range, making them suitable for incorporation into diverse food matrices without being adversely affected by internal or external factors (Calvanese et al. 2025). Especially, the incorporation of postbiotics into food products offers several distinct advantages compared to live probiotics. Postbiotics are assumed to exhibit greater stability than their viable counterparts and eliminate the need to maintain high counts of live, metabolically active cells throughout the product shelf life (Aguilar‐Toalá et al. 2018; Calvanese et al. 2025). Their thermal stability is particularly consequential for food manufacturing, postbiotics can be incorporated into products prior to heat treatment, thereby reducing the risk of secondary microbial contamination during processing (Barros et al. 2020). These advantages include: (i) minimal or no interaction with other food components, which directly translates to extended shelf stability, (ii) enhanced compatibility with diverse food processing techniques, (iii) simplified storage and transportation, including packaging and cold chain logistics, leading to longer shelf life and ease of application, with particular promise for deployment in low resource settings where maintaining controlled production and storage conditions is especially challenging, (iv) elimination of the risk of translocation from the intestinal lumen into the bloodstream, and direct beneficial effects on intestinal epithelial cells through bioactive components that may traverse the mucus layer more readily following cell inactivation, (v) substantially reduced though not entirely eliminated risk of horizontal transfer of antibiotic resistance genes, with factors such as residual DNA, strain safety, and production process control still warranting careful evaluation, and (vi) a safer alternative for immunocompromised individuals (de Almada et al. 2016; Lee et al. 2023). Furthermore, the ability to incorporate postbiotics into numerous matrices possesses the potential to diversify the functional food market by allowing integration into a much broader spectrum of products (Mudaliar et al. 2024). For instance, postbiotics can be added to non‐dairy food products with low pH and a_w_ environments that are typically unfavorable for probiotic survival. Moreover, postbiotic activity remains unaffected during the storage and transportation of these products (Kumar et al. 2023; Zhong et al. 2024). Another significant aspect is the application of postbiotics, such as EPS, peptides, and organic acids, in the development of active packaging for food bio‐preservation. The use of postbiotics is not only simpler compared to probiotics but also ensures a longer shelf life for the packaging itself. Active antimicrobial food packaging systems can utilize postbiotics through several distinct methods: (i) surface coating/adsorption: applying a thin layer of postbiotics onto the polymer surface, (ii) immobilization: binding postbiotics like bacteriocins and enzymes to polymers via ionic or covalent bonds, (iii) direct integration: incorporating postbiotics directly into the packaging polymer matrix, (iv) lamination: sandwiching an active film loaded with postbiotics between two outer layers to enhance stability and regulate metabolite migration (Calvanese et al. 2025). Also, in the formulation of fish, meat products, cheese, bakery goods, and liquid foods, postbiotics in dry form are utilized in either free or encapsulated states. Conversely, for fish and meat fillets, poultry, fruits, and vegetables, postbiotic solutions are applied as sprays or incorporated into coatings and films. While each method possesses distinct advantages and disadvantages, the selection is influenced by various factors, including the specific characteristics of the food, the form of the postbiotics, and the physical properties of the film or coating solutions (Costantini et al. 2013; Sharafi et al. 2023). Despite their inherent stability compared to live probiotics, postbiotics can still be susceptible to degradation from severe food processing conditions and rapid dilution or enzymatic breakdown during gastrointestinal transit (Karabacak Aydin et al. 2026). To address these vulnerabilities and ensure targeted delivery, encapsulation strategies have emerged as a critical focus in postbiotic research (Hosseinzadeh et al. 2025; Sharafi et al. 2023). Various advanced encapsulation techniques, including spray drying, freeze drying, and coacervation, are actively employed to enclose postbiotic metabolites within protective biopolymer matrices (Calvanese et al. 2025; Garg et al. 2026). Wall materials frequently utilized for this purpose include polysaccharides (such as alginate and chitosan) and proteins (such as whey protein and gelatin) (Awad et al. 2025; Hosseinzadeh et al. 2025). Furthermore, lipid based delivery systems, such as liposomal encapsulation and nanoemulsions, have proven highly effective in protecting postbiotic components during processing and storage. For instance, gel based liposomal systems and double cross‐linked hydrogels have been successfully applied to enhance the antibacterial efficacy of postbiotics and ensure the sustained, controlled release of specific metabolites, such as indole‐3‐propionic acid, directly into the lower gastrointestinal tract (Karabacak Aydin et al. 2026). In food applications, encapsulation preserves the bioactivity of postbiotics throughout the product's shelf life (Pawar et al. 2026). Recent studies have demonstrated that spray dried postbiotic microcapsules, such as encapsulated maltogenic amylase from *Bacillus licheniformis* , can significantly delay staling in bakery goods, while postbiotic nanoemulsions have improved the physicochemical stability and microbiological safety of dairy products like butter (Kumar et al. 2024; Nikravan et al. 2024; Pawar et al. 2026). By masking undesirable sensory attributes and shielding bioactives from harsh environments, these encapsulation strategies represent a vital technological advancement for the widespread industrial application of postbiotics (Garg et al. 2026; Karabacak Aydin et al. 2026). Additionally, the utilization of postbiotics in food technology represents a highly promising field that aligns with the principles of sustainability and resource efficiency. It holds significant potential to contribute to the reduction of chemical additives and the prevention of food waste and loss (Calvanese et al. 2025). The literature indicates that postbiotics, obtained in various formulations and ratios from diverse bacterial strains, are being integrated into a wide range of food products to serve various objectives. Current research reveals that these applications improve both functional and technological characteristics and prolong shelf life, all without compromising overall product quality. Postbiotic applications in various food matrices are summarized in Table 4.

**TABLE 4:** Food applications of postbiotics.

Source microorganism/postbiotic component	Food matrix	Application type and ratio	Key outcomes	References
*Lactobacillus brevis* TD4 postbiotics (cell‐free supernatant)	Beef slices	0%, 5%, 10%, and 15% into the edible coating	The growth of microbial agents including total viable count, psychrotrophic count, total coliform bacteria, *Escherichia coli* , *Staphylococcus aureus* , and fungi along with lipid oxidation in beef, was effectively suppressed. Application of the postbiotic preparation within the coating solution more successfully preserved the samples' pH value, moisture content, and hardness. Moreover, the postbiotic‐based edible coating was effective in maintaining meat color and sensory attributes	Abbasi et al. (2024)
*Lacticaseibacillus casei* 39 (autoclaved *L. casei* 39 cells)	Kefir	0.025 g/L	Postbiotic kefir exhibited the highest degree of protein hydrolysis (76.8%) following in vitro gastrointestinal digestion and demonstrated the highest anticancer activity against HT29 colon cancer cells	Akan et al. (2025)
*Lacticaseibacillus casei* ATCC 393 postbiotic powder (lyophilized cell‐free supernatant)	Yogurt	0.25% and 0.5%	Throughout the storage period, viable cell counts of *Streptococcus thermophilus* and *Lactobacillus delbrueckii* subsp. *bulgaricus* in the postbiotic‐enriched yogurts fell within the ranges of 8.61–9.16 log CFU/g and 7.68–9.00 log CFU/g, respectively. Yogurts fortified with postbiotic powder displayed the highest phenolic content values, while the sample containing 0.5% postbiotic exhibited the greatest total antioxidant activity. ACE‐inhibitory activity was likewise favorably affected by the inclusion of postbiotic powder. According to SDS‐PAGE analysis, no alterations were detected in either the casein or serum protein fractions as a result of postbiotic powder addition. Nevertheless, sensory scores declined slightly over the course of storage following postbiotic supplementation	Atasoy and Şengül (2026)
*Lactiplantibacillus plantarum* EPS	Tomato, Blueberry, Queso fresco cheese	EPS solution with eugenol oil in a 60:40 (v/v) ratio	A reduction of 3 log units in *Salmonella* and *E. coli* counts was achieved in tomatoes and blueberries, while a 4 log unit reduction in *L. monocytogenes* was observed in Queso Fresco cheese	Balyan et al. (2025)
*Lacticaseibacillus casei* 01 (bacterial suspension inactivated by ohmic heating)	Whey‐grape juice beverage	100 mL of bacterial suspension	The paraprobiotic, grape‐flavored whey beverage may serve as an effective alternative to its probiotic counterpart in reducing postprandial glycemic response among healthy individuals.	(Barroset al. 2021)
*E. coli* Nissle 1917 postbiotics (yogurt water extracts)	Functional yogurt enriched with cape gooseberry	2%	Postbiotics significantly increased antimicrobial, antitumor, and antioxidant activities, as well as the total phenolic content and bacterial viability	Darwish et al. (2022)
*Lacticaseibacillus paracasei* postbiotics (desalted cell‐free supernatant)	Cookie	1%, 2%, 3%, 4%, and 5%	Notable antioxidant effects, anti‐inflammatory, and antihemolytic properties antimicrobial activity, along with a marked suppression of the rise in peroxide value and malondialdehyde levels observed in cookies	Dong et al. (2024)
Heat‐killed kefir bacteria, autolyzed and dried *Saccharomyces cerevisiae*	Kefir	10^9^ and 10^10^ log CFU/mL heat‐killed kefir bacteria, 0.5% and 1% autolyzed and dried *S. cerevisiae*	In the samples enriched with postbiotics, an increase in antioxidant activity and phenolic content was observed, while no changes were detected in the basic composition or microbial characteristics. The in vitro gastrointestinal digestion results also indicate that the bioaccessibility of the antioxidant and phenolic contents in the enriched samples increased, and the antimicrobial activity was strengthened	Eminoğlu et al. (2025)
*L. plantarum* and *L. paracasei* supernatant (cell‐free fraction) and pellet (treated bacterial cells)	Fresh cheese	2%	In order to isolate and identify LAB strains and to prepare postbiotics from the selected isolates, pasteurization, sterilization, pascalization, and sonication techniques were applied, and the antibacterial properties of the resulting postbiotics were subsequently determined. Among these methods, pascalization proved to be the most effective for postbiotic preparation, with pascalization‐derived postbiotics exhibiting notable antistaphylococcal activity	Gajewska et al. (2026)
Postbiotics obtained from fermented rice bran produced by *Lactiplantibacillus* strains	Salmon fillets	Incorporation of 10% (v/v) bran into chitosan films	Enhancement of the films' mechanical and barrier characteristics, evidenced by increased tensile strength and elongation at break alongside reduced water vapor and oxygen permeability; the manifestation of antioxidant activity and capacity; and, upon application to salmon fillets, a reduction in both lipid oxidation and protein degradation together with inhibition of microbial proliferation	Ghamry et al. (2025)
Postbiotic of *Pediococcus acidilactici* (inactivated and freze‐dried cells)	Meatball	3% and 6%	The application of 6% postbiotic+0.02 M EDTA achieved a reduction of 1.32 to 3.11 log CFU/g in pathogen counts. Furthermore, this treatment resulted in significant decreases in psychrotrophs, total viable count, LAB, molds‐yeasts, and *Pseudomonas* species throughout the storage period. Characterization analyses revealed that the postbiotic preparation encompasses a broad spectrum of bioactive compounds, comprising 19 free amino acids (6.97–699.15 mg/100 g), 5 organic acids (2.15–30.64 g/100 g), 15 polyphenolic compounds (0.03–383.78 mg/100 g), short‐, medium‐, and long‐chain free fatty acids, as well as several volatile constituents, including pyrrole, pyranone, and pyrazine derivatives	İncili et al. (2023)
Postbiotics (supernatant) produced by *Lacticaseibacillus rhamnosus* and *Limosilactobacillus reuteri*	Red meat	100 mL/mg	Strong effect against *S. aureus* and *E. coli* , thereby causing a decrease in bacterial concentrations, and an increase in antioxidant activity, phenolic content, and organic acid content	Jalali, Mojgani, Haghighat, et al. (2024), Jalali, Mojgani, Sanjabi, et al. (2024)
*Liquorilactobacillus hordei* SK‐6, *Lactiplantibacillus plantarum* Y48, and *Lp. plantarum* VB‐29 cell‐free freeze‐dried postbiotics	Bread	1% and 3%	A bread formulation was supplemented with the Postbiotic contaminated with *Penicillium expansum and Aspergillus niger*, both independently and in combination with potassium sorbate, and was additionally applied as a surface spray. Notably, the formulation comprising 3% postbiotic combined with 0.1% potassium sorbate achieved complete inhibition of both *P. expansum* and *A. niger* growth	Kaya et al. (2025)
Postbiotics isolated from *Lactobacillus acidophilus* , *L. paracasei* , and *L. plantarum*	Pasteurized milk	Low minimum inhibitory concentration index = 15 mg/mL	Postbiotics can possess beneficial functional properties (antibacterial and antioxidant) and can be used as an effective preservative to inhibit *S. aureus* in milk	Khani, Shkouhian, et al. (2023)
*Lacticaseibacillus casei* 431, *Levilactobacillus brevis* ATCC, and *Limosilactobacillus reuteri* ATCC 367	Milk	Minimum inhibitory concentration = 70 μg/mL	Postbiotics may possess beneficial antimicrobial and antibiofilm properties. The prepared postbiotic exhibited a minimum inhibitory concentration (MIC) of 70 μg/mL. Within the food matrix, the minimum effective concentration (MEC) values of the postbiotics differed significantly, with the lowest MEC index (100 mg/mL) observed for the postbiotic derived from *L. brevis* . Furthermore, postbiotics obtained from *L. brevis* exhibited the highest antimicrobial activity relative to those derived from *L. reuteri* and *L. casei* . Collectively, these findings suggest that postbiotics extracted from *Lactobacillus* strains may possess functional properties namely, anti‐biofilm and antimicrobial potential both in vitro and within food model systems	Khani, Soleimani, et al. (2023)
Freeze‐dried *Lactobacillus casei*	Soy‐based milk substitutes	0.3 g	A thermal inactivation step applied at 90°C for 30 min successfully converted the probiotic soy‐based milk substitutes into a postbiotic form and preserved 62.12% of its antioxidant activity. It is stated that the resulting postbiotic product could serve as a new alternative for soy‐based functional beverages	Kongsinkaew et al. (2024)
*Lacticaseibacillus rhamnosus* GG 347 cell suspension	Flour suitable for infant consumption	5%	A significant improvement in the digestibility of protein and starch was observed as a result of fermentation. Minerals such as sodium, potassium, and magnesium increased during the fermentation process	Krishnasree et al. (2025)
Heat‐killed bacteria of *Lactobacillus casei* subsp. *casei* 327	Black and green tea kombuchas	0.2% (w/v)	The addition of postbiotics preserves product stability, and therefore, kombucha is assumed a potential food product that can serve as a postbiotic carrier	Lau and Tang (2025)
*Enterococcus durans* ED 26E/7 (concentrated substance)	Cow, goat, and sheep‐goat milk yogurts	10^7^–10^9^ CFU/mL	Growth of indicator bacteria was inhibited by 60.5% and *Staphylococci* was inhibited by 79%. Out of 46 fecal *E. coli* isolates, 40 were inhibited	Lauková, Dvorožňáková, et al. (2024)
Encapsulated *Lactococcus lactis* (strains MK 2/2, MK2/7, MK2/8)	Goat milk yogurt	0.5 g of each	Concentrated postbiotics suppressed the growth of *Staphylococci* and *Enterococci* by as much as 97.8%, achieving inhibitory activity levels of up to 800 AU/mL	Lauková, Maďar, et al. (2024)
*Lactobacillus acidophilus* LA‐5 supernatant	Ultrafiltered white cheese	200 mg/mL	The initial bacterial count of 4.46 log CFU/g was completely inactivated after 15 days of storage at 4°C using an antibacterial biopolymer‐based film formulated using chia seed mucilage in combination with postbiotics	Mardani et al. (2025)
*Lacticaseibacillus rhamnosus* CWKu‐12 cell‐free supernatant	Lamb meat	1% and 2%	Edible film produced using postbiotics and *Plantago lanceolata* mucilage, reduced the microbial load significantly compared to the control sample, and the shelf life of lamb meat was extended	Mirzaei et al. (2025)
*Lactobacillus acidophilus* and *Lactiplantibacillus plantarum* heat‐inactivated cells	Wheat germ flour	10^7^ CFU/mL	The incorporation of postbiotics resulted in a marked decrease in pH, acidity, and alcohol content during fermentation, a characteristic that could prove advantageous in applications where elevated alcohol levels are undesirable. In addition, the results demonstrated that postbiotics exerted a favorable influence on the technological attributes of the fermented product. Rheological assessment revealed that postbiotic‐containing samples possessed the highest consistency coefficient, indicative of a thicker and more stable product structure. Furthermore, the observed increase in viscosity, accompanied by shear‐thinning behavior, suggests that these strains are capable of enhancing the texture and mouthfeel of the final product. Collectively, such observations point to the capacity of postbiotic incorporation within fermentation processes to produce items with superior sensory and functional attributes	Mirzaei et al. (2026)
Inactivated with heat or sonication *Lacticaseibacillus casei* 431 and *Lactobacillus acidophilus* LA‐5	Doogh (a typical Iranian fermented milk drink)	10^7^ CFU/mL	Postbiotics can be successfully used in the production of fermented milk beverages that possess reasonable sensory attributes along with an elevated antioxidant capacity relative to the control group	Moghadam et al. (2024)
Postbiotics (supernatant) of *Limosilactobacillus fermentum*	Fish (salmon) fillets	0.5%, 1%, and 1.5%	Water vapor permeability decreased, sensory quality increased, and pH, moisture, thiobarbituric acid, and total volatile basic nitrogen values decreased	Mokhtaran et al. (2025)
Inactivated cells of *Lactobacillus acidophilus* ATCC SD 5221 and *Bifidobacterium animalis* subsp. *lactis* BB‐12	Yogurt	10^7^ CFU/L	The samples containing postbiotics had the lowest rheological parameter values, and there were significant differences among the samples in the sensory evaluation	Molaee Parvarei, Fazeli, et al. (2021)
Inactivated cells of *Lactobacillus acidophilus* ATCC SD 5221 and *Bifidobacterium animalis* subsp. *lactis* BB‐12	Yogurt	10^7^ CFU/L	The incorporation of postbiotics enhanced the viability of the starter cultures. Owing to the presence of EPS, postbiotic supplementation resulted in reduced syneresis and increased water‐holding capacity relative to probiotic yogurt samples, whereas color parameters remained unaffected by postbiotic addition	Molaee Parvarei, Fazeli, et al. (2021)
Inactivated cells of *Lactobacillus acidophilus* ATCC SD 5221 and *Bifidobacterium animalis* subsp. *lactis* BB‐12	Yogurt	10^7^ CFU/L	Samples to which postbiotics were incorporated prior to fermentation displayed a distinct structural pattern relative to the probiotic samples, characterized by larger, more open pores. In contrast, samples where probiotics and postbiotics were added after fermentation revealed a more compact structure. Yogurt samples containing neither probiotics nor postbiotics displayed a spongy structure. Due to the presence of EPS and cellular components, the sample with postbiotics added before fermentation possessed the lowest syneresis, the highest water‐holding capacity, and the highest viscosity values	Molaee Parvarei, Khorshidian, et al. (2021)
Postbiotics of (cell‐free supernatant) *Lactobacillus acidophilus* LA‐5, *L. salivarius* , and *L. casei* 431	Minced meat and full‐fat milk	10–45 mg/mL	The antibacterial activity exhibited by all postbiotics examined was primarily attributed to pyrrolo [1,2‐a] pyrazine‐1,4‐dione, with organic acids contributing to a lesser extent. Postbiotics derived from all *Lactobacillus* spp. retained more than 50% of their residual antimicrobial activity across a range of pH levels (4, 5, 6, 7, 8, and 9). Additionally, these postbiotics demonstrated biofilm‐removal activity against *L. monocytogenes* , with efficacy varying according to postbiotic type and contact duration. Within the food models tested, minimum effective concentration (MEC) values varied considerably, and notably, the lowest MEC index (15 mg/mL) was recorded for postbiotics derived from *L. salivarius* . Collectively, these findings indicate that postbiotics obtained from *Lactobacillus* spp. particularly *L. salivarius* possess applicable functional properties both in vitro and within food model systems	Moradi et al. (2019)
*Lactobacillus acidophilus* LA‐5 postbiotic powders	Low‐fat yogurt	1%	While the viability of starter cultures wasn't negatively affected, an increase in antioxidant activity was observed; over time, an open gel network with larger pores was formed, and the sensory attributes of the yogurt were preserved	Pham et al. (2024)
Inactivated cells of *Lacticaseibacillus paracasei* MIUG BL80	Fermented beverage (4% spent coffee grounds, 2% sugar, 10% freeze‐dried sourdough from whole rye flour, 15% postbiotic, and 0.2% starter yeast)	15%	The resulting fermented product displayed notable bioactive characteristics, including a total titratable acidity of 13.48 mL, antioxidant activity of 2.30 mM TE/mL, and antimicrobial effects against *Bacillus subtilis* (12.00 mm inhibition zone), as well as inhibitory activity against *Staphylococcus aureus* and *Aspergillus niger*. In addition, the fermented beverage was found to contain 102.72 μg/mL gallic acid, 34.48 μg/mL protocatechuic acid, 8.62 μg/mL caffeine, and 3.89 μg/mL 4‐hydroxybenzoic acid alongside vanillic acid and cafestol. Consumer perception assessments further indicated favorable average scores across key evaluated attributes	Pihurov et al. (2026)
Concentrated CFS obtained from *Lacticaseibacillus rhamnosus* GG and *Lactobacillus acidophilus*	Chicken breast meat	500 μL/mL; 5:1 ratio	Both postbiotics significantly improved meat quality by lowering the product's pH, TBARS values, and drip loss, while increasing the water‐holding capacity compared to the control sample. The postbiotics preserved the product's color, resulting in higher a* and b* values, and improved the texture of both raw and cooked meat. While LGG postbiotics exhibited superior antioxidant effects, LA postbiotics had a more positive impact on the sensory attributes of the product	Rahman et al. (2025)
*Lactobacillus acidophilus* ATCC 4356, *Lactiplantibacillus plantarum* NBRC 3070, *Lacticaseibacillus rhamnosus* GG ATCC 53103 and *Lacticaseibacillus casei* ATCC 393CFS	Vacuum packaged broiler breast meat	1:5 ratio in the meat model	Postbiotic treatments conferred more pronounced early‐stage suppression, reducing these pathogens by 80%–86%, with LGG‐derived postbiotics exhibiting the greatest efficacy. These treatments decreased total mesophilic aerobic bacteria, Gram‐positive and Gram‐negative pathogens to 1.06–3.64, 0.93–3.30 and 1.41–4.36 CFU/g, respectively. Notably, LGG postbiotics achieved a greater reduction in *Salmonella typhimurium* levels in broiler meat compared with those obtained using *Lactobacillus acidophilus* and *Lactiplantibacillus plantarum*	Rahman et al. (2026)
*Lactiplantibacillus plantarum* UCLM56 cell‐free extract	Sheep milk yogurt	20 mL	Yogurt enriched with postbiotic extract showed an increase of more than 360% and 260% in GABA and propionic acid levels, respectively, compared to traditional yogurt, along with an enhanced antioxidant capacity. Following in vitro fermentation, the yogurt sample with postbiotic addition exhibited significant increases in lactic and propionic acid levels by 50% and 41%, respectively and more than 200% improvement in antioxidant capacity	Ramos et al. (2025)
Postbiotics derived from *Streptococcus thermophilus* and *Lactobacillus delbrueckii* subsp. *bulgaricus*	Yogurt	3%	Among the samples tested, the whey sample demonstrated the highest antioxidant activity, corresponding to 18.71% inhibition, and likewise exhibited the greatest water‐holding capacity, reaching 77.93% and a tendency to receive the highest scores in sensory evaluation compared to other samples. Yogurt samples produced with the addition of postbiotics derived from *Lactobacillus delbrueckii* subsp. *bulgaricus* exhibited significantly better texture properties compared to those produced with postbiotics derived from *Streptococcus thermophilus*	Sadighbathi et al. (2023)
Postbiotic solution of *Lactiplantibacillus plantarum*	Rainbow trout fillets	Spray fillets at 1 mL/10 g trout meat	The shelf life of the fillets can be extended by more than 3 days, and the sensory characteristics and quality parameters are positively affected	Sharafi et al. (2024)
*Lactobacillus bulgaricus* postbiotic solution	Sausage	150 mg/L and 300 mg/L	The sample coated with 300 mg/L postbiotic in combination with 1% chitosan generally satisfied the target parameters for pH, total nitrogen, fat, and moisture content. Furthermore, these coated samples demonstrated the most pronounced inhibitory effect against psychrophilic and mesophilic bacteria, as well as yeasts and molds	Sheikhi et al. (2025)
*Lacticaseibacillus rhamnosus* SD11 and *Lacticaseibacillus paracasei* SD1 postbiotics (heat‐killed cells)	Fermented milk tablets	4 × 10^6^ CFU/mL	Fermented milk tablets containing Postbiotics effectively suppressed the proliferation of pathogenic microorganisms, reduced pro‐inflammatory cytokine concentrations, inhibited Caco‐2 cell growth, and promoted butyrate synthesis. Postbiotic tablets maintained heat‐inactivated cells at a level of 4 log CFU/g for 6 months	Teanpaisan et al. (2024)
*Lactobacillus acidophilus* postbiotics (secondary metabolites and inanimate cells)	Soft cheese	5% and 10%	The cheese samples containing postbiotics are more stable, with no changes observed in bacterial counts throughout the 24‐day storage period. Furthermore, these samples were found to be free of psychrophilic bacteria, coliform bacteria, yeast, and molds	Wahhab and Al‐Mosowy (2024)
*Bifidobacterium animalis* subsp. *lactis* BB12 postbiotic powder	Low‐fat yogurt	1%	Postbiotics prepared using skim milk substantially enhanced the antioxidant capacity of yogurt without compromising its key physical and sensory attributes	Yousefvand et al. (2024)
*Lactiplantibacillus plantarum* inactivated bacterial cells	Soy‐based milk substitute	10^8^ and 10^9^ CFU/mL	The addition of postbiotics significantly increased the protein (3.28%), fat (2.35%), total phenolic content (834.32 mg GAE/g), antioxidant activity (75.44 μmol TE/g), and ash content (0.57%). In the sensory analysis, the beverage supplemented with postbiotics received the highest scores for taste and overall impression	Zahidah et al. (2024)
*Lactiplantibacillus plantarum* postbiotic solution	Sausage	−100% postbiotic −1% chitosan+100% postbiotic −0.5% chitosan+100% postbiotic	Postbiotics generated notable inhibition zones of 17 mm and 15 mm against *Salmonella typhimurium* and *E. coli* , respectively, and effectively prevented biofilm formation, with inhibition levels ranging from 9% to 46%	Zeraat pisheh et al. (2025)

Furthermore, it has been reported that probiotic cell lysates may contain sphingomyelinase, hyaluronic acid, lipoteichoic acid, EPS, lactic acid, diacetyl, peptidoglycan, and acetic acid. These components offer a broad spectrum of biological activity that can be utilized to provide various dermatological benefits, such as the improvement of skin conditions like atopic eczema and atopic dermatitis, the treatment of burns and scars, skin regenerative properties, the reinforcement of the skin's innate immunity, and protection against photo damage (Kober and Bowe 2015; Lew and Liong 2013). Numerous studies and patents have been published regarding the use of probiotic extracts, specifically postbiotics, in the development of advanced skincare formulations (Callewaert et al. 2017; Holz et al. 2017; Huang and Tang 2015).

**FIGURE 6:**
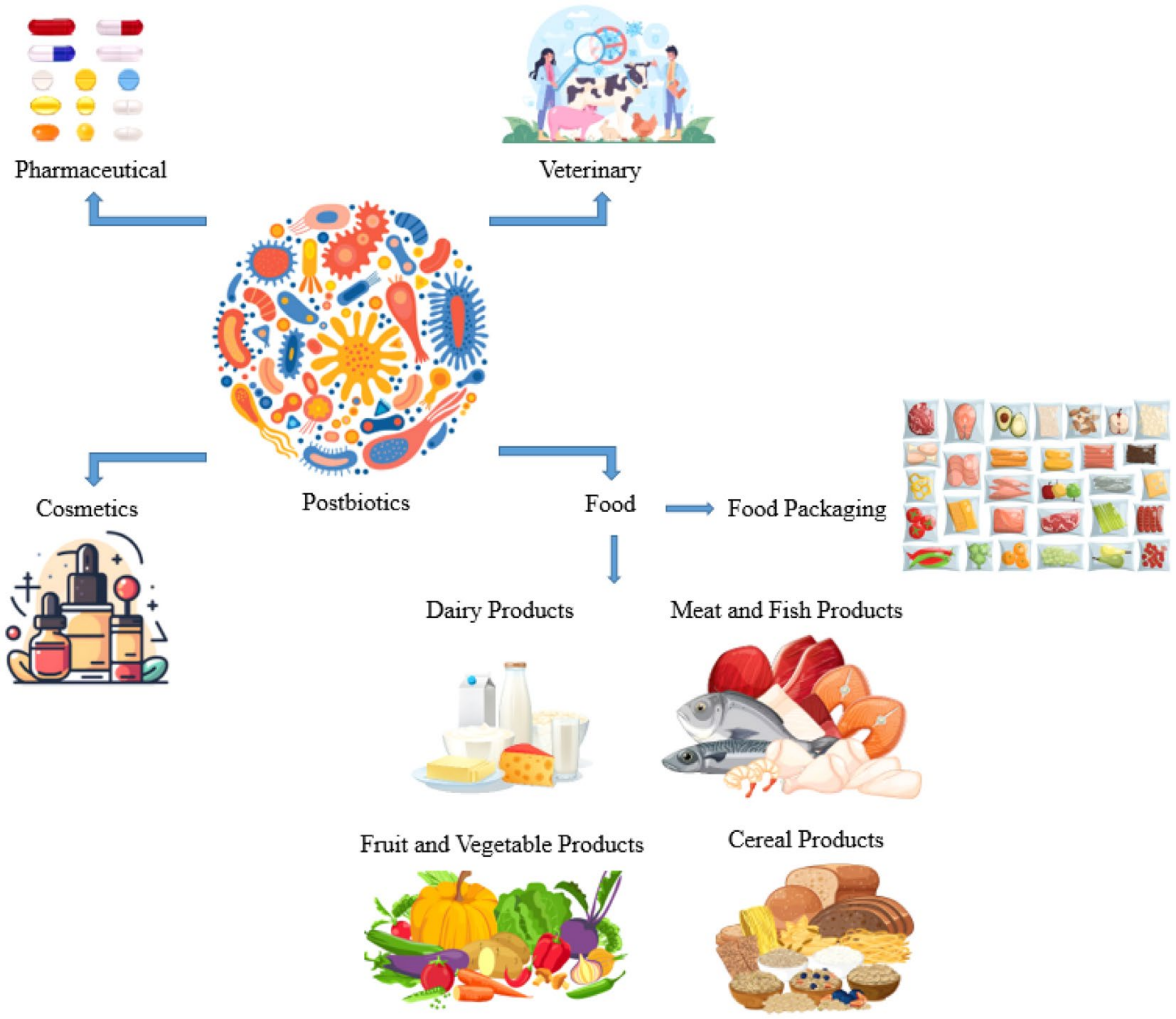
Postbiotic applications in various industries.

Another promising field of application has emerged with reports indicating that postbiotics can positively influence animal health and the growth performance of poultry and piglets (Choe et al. 2012; Kareem et al. 2016; Loh et al. 2013, 2014; Thu et al. 2011). The potential applications of postbiotics in diverse industrial and applied fields are presented in Figure 6 through a holistic framework. It reveals that postbiotics are positioned as strategic biotechnological components in areas including health, food safety, food engineering, sustainable production, and cosmetic innovation.

## 8 Health Benefits of Postbiotics

In recent years, numerous studies have been conducted using in vitro and in vivo models to evaluate the potential bioactivity and health related impacts of various postbiotics (Aguilar‐Toalá et al. 2018; Robles‐Vera et al. 2017). It has been reported that postbiotics provide health benefits similar to probiotics and may serve as a safer alternative in cases where the use of live probiotic bacteria is contraindicated. The mechanisms by which postbiotics alleviate diseases vary depending on the diversity of the postbiotic type, the health status of the host, and the specific production methods employed (Zhong et al. 2024). To translate these potential advantages into tangible health outcomes, it is essential to determine the optimum dosage, frequency of use, and duration of administration through well designed, large scale human randomized controlled trials (Baheti et al. 2026; Calvanese et al. 2025). The utilization of postbiotics reduces the risks associated with: (i) the development of opportunistic infections (such as bacteremia, fungemia, sepsis, and endocarditis), (ii) heightened inflammatory responses to vaccines or allergens (e.g., arthritis, fever), (iii) harmful metabolic effects resulting from mucus degradation and the production of deconjugated bile salts and D‐lactate which can lead to cholestasis, gastrointestinal disorders, D‐lactic acidosis, and colorectal cancer (iv) the horizontal transfer of antibiotic resistance genes to other commensal or pathogenic bacteria within the gastrointestinal tract, (v) microbial translocation. Furthermore, postbiotics maintain their bioactivity when administered in conjunction with antibacterial and antifungal agents (Aguilar‐Toalá et al. 2018; Barros et al. 2020; Taverniti and Guglielmetti 2011).

Postbiotics are thought to exert their effects through five proposed mechanisms of action: (i) modulation of the existing microbiota; this occurs through the action of lactic acid, SCFAs, bacteriocins, and adhesins, (ii) enhancement of epithelial barrier functions; the integrity of the intestinal barrier is strengthened via SCFAs, proteins, and EPS, (iii) modulation of immune responses; this mechanism involves microbe associated molecular pattern (MAMP) and pattern recognition receptor (PRR) interactions, as well as the pathways of indole derivatives, keto acids, and branched‐chain fatty acids, (iv) modulation of metabolic responses; systemic metabolism is influenced through bile salt hydrolase activity, succinate, vitamins, and short chain fatty acids, (v) systemic signaling via the nervous system; postbiotics communicate through neuroactive compounds such as serotonin, dopamine, acetylcholine, and GABA (Calvanese et al. 2025; Salminen et al. 2021). While the fundamental pathways of these five mechanisms have been extensively elucidated and validated through robust in vitro evidence and animal evidence, confirming these specific molecular pathways directly through human evidence remains challenging due to the complexity of conducting mechanistic studies in human subjects (Aggarwal et al. 2022; Calvanese et al. 2025).

Unless postbiotics can create permanent changes in the intestinal microbiota, unlike probiotics, which multiply and colonize the gut to provide long term beneficial effects, their health benefits will remain limited to the duration of their administration. In this context, although postbiotics possess the potential to correct intestinal dysbiosis and provide long term health benefits by regulating the growth of different microbial populations, the lack of sufficient experimental evidence, particularly robust and long term human evidence, makes it difficult to reach a definitive conclusion and indicates the need for further research in this field (Karabacak Aydin et al. 2026; Mudaliar et al. 2024).

### 8.1 Immunomodulation

Probiotics play a critical role in consumer health, particularly in the regulation of the immune system. Similar to probiotics, postbiotics can also exert effects on the immune system, and various studies in the literature document their immunomodulatory impacts (de Almada et al. 2016; Miyazawa et al. 2011). While clinical trials provide human evidence of reduced respiratory and gastrointestinal infection rates, the precise receptor ligand interactions and immunomodulatory pathways are primarily characterized through in vitro evidence using human and murine macrophage or dendritic cell lines, alongside animal evidence from murine models (Baheti et al. 2026; Cuevas‐González et al. 2020; Ding et al. 2026). The immunomodulatory potential of postbiotics appears to be associated with several structural components. Specifically, elements such as teichoic acid, peptidoglycan, DNA, cell surface proteins, and cell wall polysaccharides are reported to contribute significantly to strengthening the immune system and supporting the mucosal structure and barrier function of the gastrointestinal tract (Divsalar et al. 2025; Hosseini et al. 2024). Mechanistically, these structural components act as microbe‐associated molecular patterns (MAMPs) that are recognized by host pattern recognition receptors (PRRs), such as toll‐like receptors (TLRs). This receptor binding tightly regulates intracellular signaling pathways (e.g., NF‐κB and MAPK), which promotes the differentiation of regulatory T cells (Tregs) and balances Th1/Th2 immune responses by suppressing pro‐inflammatory cytokines and stimulating anti‐inflammatory mediators (Aggarwal et al. 2022). Furthermore, the synthesis of heat shock proteins (Hsp) during thermal processing is observed to enhance immunomodulatory activity demonstrated via in vitro macrophage assays and animal evidence showing enhanced innate responses (de Almada et al. 2016; Fujiki et al. 2012).

### 8.2 Antagonistic Activity Against Intestinal Pathogens

Postbiotics also possess the ability to defend the host against infections. The primary mechanism underlying the antimicrobial efficacy of postbiotics is the prevention of pathogen colonization through cell wall components and various immunomodulatory effects, which have been extensively modeled in in vitro cell adhesion assays and corroborated by animal evidence showing decreased pathogen translocation and survival (Divsalar et al. 2025; Ikram et al. 2024). Furthermore, studies in the literature have identified that they exhibit direct antimicrobial activity against pathogens via postbiotic metabolites (Divsalar et al. 2025; Salminen et al. 2021). This direct bacteriostatic and bactericidal capacity is overwhelmingly supported by robust in vitro evidence demonstrating the efficacy of cell‐free supernatants, bacteriocins, and organic acids against various Gram‐positive and Gram‐negative pathogens (Aggarwal et al. 2022; Divsalar et al. 2025; Prajapati et al. 2023).

### 8.3 Modulation of the Intestinal Microbiota

It has been stated that postbiotics exhibit restorative effects on the gut microbiota by suppressing the growth of harmful intestinal bacteria and promoting the proliferation of beneficial taxa, as seen in both animal evidence and human fecal fermentation models (Calvanese et al. 2025; Franco 2024; Ikram et al. 2024). While the underlying mechanism of action has not yet been fully elucidated, it has been observed, for instance, that the growth of hydrogen sulfide (H_2_S) producing bacteria in the gut is inhibited. It is reported that further clarification is needed regarding how postbiotics exert these effects, specifically whether they adhere to intestinal cells or form co‐aggregates with pathogens phenomena currently observed primarily as in vitro evidence (de Almada et al. 2016; Kimoto‐Nira et al. 2009; Ma et al. 2023).

### 8.4 Healing of Intestinal Injuries

It has been reported that postbiotics contribute to reduced inflammatory responses and improved intestinal epithelial barrier function, and attenuate intestinal damage through the induction of Hsp (de Almada et al. 2016; Ueno et al. 2011). This protective capacity is heavily substantiated by animal evidence, particularly in murine models of DSS induced colitis, where postbiotic administration significantly reduces histological damage and disease severity, though direct human evidence for these specific healing pathways remains an area requiring further clinical exploration (Baheti et al. 2026; Ding et al. 2026).

### 8.5 Reduction of Bacterial Translocation and Protection of the Intestinal Barrier

Postbiotics contribute to the preservation of the intestinal barrier by regulating intestinal permeability and limiting bacterial translocation. These benefits appear to be associated with certain structural components of the postbiotics, most likely the bacterial cell wall. Therefore, it is thought that the mechanism operates through cellular structures rather than metabolic activities. This hypothesis has been validated by animal evidence showing reduced translocation of bacteria to extra intestinal organs in murine models of intestinal obstruction (de Almada et al. 2016; Generoso et al. 2011). Mechanistically, structural postbiotics such as surface layer proteins (SLPs) and EPS upregulate the expression of critical tight junction proteins (e.g., ZO‐1, claudins, and occludin), effectively sealing paracellular spaces against pathogen and endotoxin infiltration (Ding et al. 2026; Gu et al. 2023). Concurrently, specific postbiotic proteins like p40 inhibit epithelial cell apoptosis and stimulate mucin secretion, further reinforcing the physical defense of the mucosal shield (Baheti et al. 2026; Gu et al. 2023; Ikram et al. 2024). The understanding of these molecular mechanisms is largely derived from in vitro evidence utilizing human colonic epithelial cell lines and animal evidence, emphasizing the need to translate these findings into definitive human evidence through clinical trials evaluating barrier integrity in patients with leaky gut syndrome (de Almada et al. 2016; Ding et al. 2026; Ikram et al. 2024).

### 8.6 Treatment of Diarrhea

Postbiotics are utilized in the treatment of chronic diarrhea to alleviate symptoms such as bloating and abdominal pain. Furthermore, postbiotics have been shown to mitigate symptoms in patients suffering from irritable bowel syndrome (IBS) in human studies. The mechanism of action for postbiotics appears to be associated with the coating of the colonic mucosa, thereby providing protection against the adhesion and invasion of pathogenic microorganisms. However, this mechanistic understanding is primarily derived from in vitro evidence (de Almada et al. 2016; Tarrerias et al. 2011).

### 8.7 Modulation of Inflammation

Postbiotics are as effective as probiotic strains in regulating inflammation induced by LPS, a finding extensively documented through in vitro and animal evidence. It is suggested that the modulation provided by postbiotics may be dependent on cellular components such as lipoteichoic acid, peptidoglycan, and cell wall. However, further studies are required to fully elucidate the precise mechanism mediating this effect in humans. Additionally, postbiotics have been shown to regulate the inflammatory response triggered by flagellin in Caco‐2 intestinal epithelial cells. Interleukin‐8 (IL‐8), a proinflammatory cytokine, has been utilized as a marker for evaluating the resulting inflammation. Postbiotic *Lacticaseibacillus rhamnosus* GG has been found to modulate the inflammatory response by reducing IL‐8 production in the intestinal epithelium (de Almada et al. 2016; Li et al. 2009; Lopez et al. 2008).

### 8.8 Mitigation of Lactose Intolerance

Postbiotics have been shown to be effective in alleviating certain symptoms associated with lactose intolerance in children, providing valuable human evidence for their therapeutic use in pediatric populations (Lee et al. 2023; Rampengan et al. 2010).

### 8.9 Reduction of Cholesterol

The hypocholesterolemic effects exhibited by postbiotics may be attributed to several mechanisms elucidated primarily through in vitro and animal evidence: (i) the degradation of beta‐glucan in the postbiotic cell wall due to the sonication process, which may be correlated with a reduction in serum cholesterol levels, (ii) the release of specific cytoplasmic components from paraprobiotics following cell inactivation, (iii) the inhibition of cholesterol synthesizing enzymes by postbiotic metabolic products, thereby reducing endogenous cholesterol production, (iv) the capacity of postbiotic metabolites to bind cholesterol, thus preventing its absorption by the body, (v) the interference of these metabolites with the recycling of bile salts, a metabolic byproduct of cholesterol, thereby facilitating their excretion (de Almada et al. 2016; Shin et al. 2010). While these mechanisms are well established in preclinical settings, comprehensive human evidence is still required to confirm their clinical efficacy (Mokashe et al. 2026).

### 8.10 Alleviation of Respiratory Disease Symptoms

Postbiotics can contribute to improving the quality of life of patients by alleviating the symptoms of respiratory diseases such as asthma, the common cold, pneumonia, influenza, and allergic rhinitis (Lee et al. 2023).

### 8.11 Improvement in Alcohol‐Induced Liver Diseases

Postbiotics have been shown to prevent the elevation of serum aspartate aminotransferase (AST) and alanine aminotransferase (ALT) levels caused by alcohol consumption, as well as the increase in total cholesterol and triglyceride levels in the liver (de Almada et al. 2016; Segawa et al. 2008). Currently, this hepatoprotective capacity is supported predominantly by animal evidence utilizing rodent models of alcohol‐induced liver injury, highlighting the need for future human clinical trials (López‐Almada et al. 2024).

### 8.12 Inhibition of Cancer Cell Growth

Postbiotics, through their various components such as intact cells, cell walls, peptidoglycan, and cytoplasmic fractions, have demonstrated anti‐proliferative activity and pro‐apoptotic effects on gastric and colorectal cancer cell lines. Nevertheless, the specific bacterial component responsible for the more pronounced inhibition of cell proliferation remains to be determined and the translation of these in vitro findings into definitive animal evidence and human evidence remains an ongoing challenge (de Almada et al. 2016; Orlando et al. 2012).

### 8.13 Treatment of Atopic Dermatitis

Postbiotics have contributed to the reduction of symptoms in adult patients with atopic dermatitis. Postbiotic treatment has been shown to decrease the severity of skin lesions in clinical settings (de Almada et al. 2016; Kim et al. 2013).

### 8.14 Modulation of the Response to Visceral Pain

Postbiotics have been found to have the potential to inhibit visceral pain induced by colorectal distension, an effect that has been primarily demonstrated through animal evidence utilizing rodent models (de Almada et al. 2016; Kamiya et al. 2006).

### 8.15 Alleviation of Colitis

Certain postbiotics have been shown to exert beneficial effects in the prevention of colitis or the alleviation of its symptoms, which is widely supported by animal evidence using chemically induced murine colitis models (Abbasi et al. 2022). Postbiotics have shown anti‐inflammatory effects on the peripheral blood mononuclear cells of patients with ulcerative colitis (de Almada et al. 2016; Imaoka et al. 2008).

### 8.16 Suppression of Certain Age‐Related Symptoms

Postbiotics have been stated to exhibit potential effects in alleviating several age‐related symptoms, such as decreased bone density, the formation of skin ulcers, and hair loss findings that are currently based on animal evidence. Furthermore, they have been shown to enhance the production of IFN‐ɣ and IL‐12, which is attributed to their ability to improve the Th1/Th2 balance. This immunomodulatory shift toward a Th1 response is crucial for enhancing cellular immunity and may play a significant role in the anti‐aging and regenerative properties observed in these preclinical models, highlighting the need for translation into long‐term human evidence (de Almada et al. 2016; Kimoto‐Nira et al. 2009).

### 8.17 Oral Health Benefits

Postbiotics have been reported to have the potential to modulate the oral microbiota and, through this mechanism, may be effective in preventing dental caries and improving oral health, a capacity supported by in vitro biofilm assays (de Almada et al. 2016; Tanzer et al. 2010).

The aforementioned health benefits indicate that postbiotics do not merely target the gastrointestinal system; they also demonstrate significant potential for use in food applications designed to achieve positive physiological effects in various other regions of the body (de Almada et al. 2016).

Postbiotics derived from a wide range of bacterial sources have been produced in various forms for use in the treatment of different diseases or for supporting various physiological systems, and commercially available products are listed in Table 5.

**TABLE 5:** Commercial postbiotic products.

Product	Company	Postbiotic content	Intended use
Lacteol Lacteol Baby Lacteol ORS	Carnot Laboratories, France	*Limosilactobacillus fermentum*, *L. delbrueckii*, *L. acidophilus* LB	Treatment of diarrhea
Pylopass	Novozymes Berlin GmbH, Germany	*Limosilactobacillus reuteri* DSM 17648	Treatment of *Helicobacter pylori* infection
Del‐Immune V	Stellar Biotics, France	*L. rhamnosus* V	Treatment of diarrhea and constipation, Immunological support
LAC‐Shield	Morinaga Probiotics Center, Japan	*L. paracasei* MCC1849	Immunological support
Staimune‐BC30	Ganeden, America	*B. coagulans* GBI‐30	Immunological support
L‐137	MCLS EUROPE, Holland	*L. plantarum* L‐137	Immunological support
Colimil Baby	Humana, Spain	*L. acidophilus* HA122	Treatment of colic
Bactistatin	Kraft, Russia	*Bacillus subtilis* VKPM V‐2335	Gastrointestinal support
CytoFlora	BioRay Inc., America	*Lacticaseibacillus rhamnosus, Bifidobacterium longum, Bifidobacterium infantis, Bifidobacterium bifidum, Lactobacillus acidophilus, Lactobacillus reuteri, Lactiplantibacillus plantarum, Lactobacillus casei, Lactobacillus paracasei Lactobacillus helveticus, Lactobacillus salivarius*	Gastrointestinal support, Immunological support
Hylak Forte	Ratiopharm/Merckle, Germany	*Escherichia coli* DSM 4087, *Streptococcus faecalis* DSM 4086, *Lactobacillus helveticus* DS 4183, *Lactobacillus acidophilus* DSM 414	Gastrointestinal support
Pro‐Symbioflor	SymbioPharm, Germany	*Enterococcus faecalis* DSM 16440 *Escrerichia coli* DSM 17252	Gastrointestinal support, Immunological support, Atopic dermatitis treatment
Nyaditum resae	Manremyc S.L., Spain	*Mycobacterium manresensis*	Tuberculosis treatment
Colibiogen	Laves‐Arzneimittel GmbH, Schötz, Switzerland	*Escherichia coli* (strain laves 1931)	Pathogen inhibition, Treatment of skin lesions
Immuse	Kirin and Kyowa Hakko, Japan	*Lactococcus lactis*	Immune system activator

The products summarized in Table 5 differ substantially with respect to regulatory classification, intended health claims, target populations, dosage regimens, and the level of supporting clinical evidence. Most commercially available postbiotic preparations are marketed as dietary supplements, functional food ingredients, or medical foods, whereas a limited number of products are registered as medicinal products in certain jurisdictions (de Simone 2019; Vinderola et al. 2023). The active components of these formulations primarily consist of heat‐inactivated microbial cells, cellular fragments, fermentation‐derived metabolites, or combinations thereof (Guglielmetti et al. 2025; Pimentel et al. 2023). Recommended dosages vary considerably depending on the specific formulation and intended application, ranging from milligram quantities of inactivated biomass to preparations containing several billion non‐viable microbial cells per daily dose (Calvanese et al. 2025; Mokashe et al. 2026; Singh et al. 2025).

The health claims associated with these products can be broadly categorized into gastrointestinal, immunomodulatory, anti‐infective, and disease specific applications (Calvanese et al. 2025; Mokashe et al. 2026). While certain formulations are intended for the management of acute gastrointestinal disturbances others have been primarily developed to support immune function or to serve as adjuncts to conventional therapeutic strategies (Szajewska and Salminen 2023; Vinderola et al. 2023). Target populations encompass infants, children, healthy adults, elderly individuals, and specific patient subgroups, depending on the clinical indication addressed (Calvanese et al. 2025; Vinderola et al. 2022).

The strength of the scientific evidence underlying these products remains markedly heterogeneous (Calvanese et al. 2025; Mokashe et al. 2026). Selected postbiotic strains are supported by randomized controlled trials demonstrating measurable physiological benefits (Calvanese et al. 2025; Vinderola et al. 2022). In contrast, the majority of other products rely predominantly on preclinical findings, observational data, or limited clinical investigation (Mokashe et al. 2026). Consequently, the overall level of evidence ranges from low to high depending on the specific product and the health outcome claimed (Szajewska and Salminen 2023; Vinderola et al. 2022).

Based on currently available information, the majority of products presented in Table 5 may reasonably be classified as postbiotics, as they contain deliberately inactivated microorganisms or their derived bioactive constituents (Cuevas‐González et al. 2020; Pawar et al. 2026). Nevertheless, for several formulations, the depth of physicochemical characterization and the robustness of clinical efficacy data remain insufficient, underscoring the continued need for standardized production protocols, mechanistic investigation, and well‐designed clinical trials to substantiate their health claims and clarify their regulatory positioning (Cuevas‐González et al. 2020; Guglielmetti et al. 2025; Mokashe et al. 2026).

## 9 Challenges and Future Perspectives for the Application of Postbiotics in Food Products

Even though postbiotic applications in food technology are generally promising, more comprehensive research is required to address several challenges hindering their widespread use. The primary challenge regarding inactivated cells is the ambiguity in their definition and the lack of precise information concerning the type and quantity of their bioactive compounds (Divsalar et al. 2025). The lack of a globally harmonized and universally accepted definition has created significant confusion among scientists, industry professionals, and regulatory bodies. While the ISAPP proposed a consensus definition requiring the presence of inanimate microorganisms and/or their components, discrepancies remain in the literature regarding whether purified metabolites or CFS alone can be classified as postbiotics (Divsalar et al. 2025; Guglielmetti et al. 2025; Salminen et al. 2021). While it is stated that these cells offer health benefits, much of the literature suggests that these advantages are directly linked to consumption. However, determining the true clinical significance of these benefits remains challenging. Current evidence relies heavily on in vitro and animal models, and there is a critical scarcity of large scale, well designed human randomized controlled trials to validate their long term efficacy, establish specific molecular mechanisms of action, and substantiate definitive health claims in human populations (Calvanese et al. 2025; Mokashe et al. 2026).

Although various inactivation methods have been employed to obtain postbiotics, these methods have not been standardized due to the vast diversity of strains (Lee et al. 2023). Since the inactivation of probiotics through different techniques can lead to variations in the characteristics and subsequent health benefits of the resulting postbiotics, further research is required to compare and establish standardized parameters for postbiotic production. Without such standardization, achieving batch to batch reproducibility at an industrial scale is extremely difficult, as variations in fermentation, extraction, and scaling‐up processes can drastically alter the final metabolite profile and bioactivity of the preparation (Amobonye et al. 2025; Garg et al. 2026). Furthermore, the selection of target microorganisms necessitates the use of specific criteria and sensitive methodologies to determine the biological activity of the inactivated cells. A critical part of this analytical challenge involves live cell testing and viability assessment. Traditional plate counting often fails to accurately differentiate between dead, damaged, and VBNC, necessitating the adoption of advanced culture independent techniques like flow cytometry and qPCR to ensure product consistency and verify complete inactivation (Boyte et al. 2023; Guglielmetti et al. 2025). Another critical consideration is the need to evaluate the efficacy and sustainability of postbiotics within the intestine, given their lack of replicative capacity (Mudaliar et al. 2024). This lack of replication also complicates dosing strategies; unlike live probiotics, postbiotics cannot colonize the gut, making it imperative to establish the minimum effective concentration and standardize dosage protocols to ensure that the administered amount translates to reproducible physiological benefits (Mokashe et al. 2026; Pawar et al. 2026).

In food applications, potential physical and chemical changes following the addition of postbiotics are a matter of concern, as some postbiotic mixtures exhibit high moisture content, a brown or yellowish appearance, or specific sensory characteristics (Divsalar et al. 2025; Sharafi et al. 2023). Such sensory effects, including strong acidic, sour, or vinegary off‐flavors resulting from short chain fatty acids or the yellowish‐brown discoloration from residual synthetic culture media like MRS broth, can severely compromise consumer acceptability (Divsalar et al. 2025; Pawar et al. 2026). Moreover, incorporating postbiotics may modify the taste and aroma profile of food products, owing to the presence of acidic constituents as well as the intrinsic flavor characteristics of the culture medium. It is essential to utilize suitable methodologies to determine their biological effects and examine potential changes in the color, texture, and sensory properties of the food, and evaluate the stability and activity of postbiotics throughout the shelf life of food products (Divsalar et al. 2025; Sharafi et al. 2023). This evaluation must strictly account for interactions within the food matrix, as the antimicrobial and bioactive efficacy of postbiotics can be significantly diminished when components bind to food lipids, proteins, or carbohydrates, or are degraded by endogenous enzymes and pH fluctuations during processing and storage (Pawar et al. 2026; Sharafi et al. 2023). Other major issues that must be considered include the impact of proteolytic and lipolytic enzymes, which may remain at high levels when thermal processing is not applied, and the lack of safety and regulatory standards in postbiotic production (Divsalar et al. 2025; Sharafi et al. 2023). Given the importance of this topic for the practical applicability of postbiotics in food systems, the absence of a unified global regulatory framework severely impedes their commercialization. This lack of harmonization leads to fragmented approval processes where postbiotics must be distinctly classified either as food ingredients, novel foods, or health supplements, depending on the jurisdiction (Guglielmetti et al. 2025; Vinderola et al. 2023). In the European Union, postbiotics are subjected to the stringent Novel Food Regulation if the progenitor microorganism lacks a history of safe consumption prior to 1997. For instance, pasteurized *Akkermansia muciniphila* recently had to undergo rigorous toxicological assessments by the European Food Safety Authority (EFSA) to be authorized as a novel food (Mokashe et al. 2026; Vinderola et al. 2023). In the United States, the Food and Drug Administration (FDA) regulates postbiotics based on their intended application, requiring them to attain Generally Recognized as Safe (GRAS) status for use as conventional food ingredients or to be submitted as New Dietary Ingredients (NDIs) if marketed as dietary supplements (Akter et al. 2026; Garg et al. 2026; Guglielmetti et al. 2025). Conversely, in Japan, postbiotics, traditionally termed “biogenics”, have a long history of use and can be registered more seamlessly under the Foods with Function Claims (FFC) system provided they meet specific evidentiary requirements (Akter et al. 2026; Guglielmetti et al. 2025; Pimentel et al. 2023). This fragmented regulatory landscape highlights critical gaps between existing frameworks and the specific needs of postbiotic products. A major gap lies in the safety evaluation process; regulatory bodies often rely on the Qualified Presumption of Safety (QPS) or GRAS status of the live progenitor strain. However, the safety of a live strain does not automatically guarantee the safety of its inanimate counterpart, as cell lysis during inactivation, particularly of Gram‐negative bacteria, can release LPS and endotoxins that may induce severe inflammatory responses or toxic shock in vulnerable populations (Divsalar et al. 2025; Thorakkattu et al. 2022). Furthermore, securing health claims for postbiotics requires robust scientific substantiation. Regulators like EFSA demand high quality, randomized, double blind, placebo controlled human trials to establish clear cause and effect relationships for health claims, treating postbiotics with evidence requirements more akin to pharmaceutical agents than conventional foods (Guglielmetti et al. 2025; Pristavu et al. 2022). Therefore, establishing customized regulatory frameworks that define precise criteria for endotoxin limits, compositional profiling, and standardized viability assays is urgently needed to bridge the gap between scientific innovation and safe industrial implementation (Guglielmetti et al. 2025; Moghadam et al. 2026). Beyond regulatory approval, manufacturers face difficulties in determining the optimal dosage and frequency of use, and problems related to the labeling and the quantification of the composition of postbiotic products (Divsalar et al. 2025). Proper labelling presents its own set of hurdles, requiring manufacturers to transparently declare the progenitor strain's identity, the exact nature of the postbiotic preparation, the inactivation method, and accurately quantified active units to ensure compliance and build consumer trust (Guglielmetti et al. 2025; Mokashe et al. 2026). To address these issues, a viable strategy involves shortening the contact duration between postbiotics and the food matrix, or alternatively, employing lower postbiotic concentrations alongside other potential antimicrobial agents (Moradi et al. 2021; Sharafi et al. 2023; Zhong et al. 2024).

## 10 Conclusion

This review demonstrates that postbiotics have evolved from a conceptual extension of probiotic science into a multidimensional field at the intersection of microbiology, food engineering, and regulatory science. Translating this scientific momentum into reliable, commercially viable food products, however, requires that several interconnected gaps be systematically addressed. Standardization remains the most pressing limitation across the field. Future efforts should therefore prioritize the development of strain‐specific, outcome‐linked inactivation guidelines that are extended systematically to non‐thermal technologies. Beyond standardization, analytical validation must also progress beyond traditional viability assessment to ensure consistent and mechanistically informed characterization of postbiotic preparations. Equally important, the health benefits attributed to postbiotics remain predominantly supported by in vitro and preclinical evidence, underscoring the need for well designed, adequately powered human clinical trials to substantiate these claims and strengthen their scientific credibility. Regulatory alignment likewise constitutes a critical bottleneck, as postbiotics are currently regulated inconsistently and classified as dietary supplements, medical foods, or medicinal products depending on jurisdiction. Harmonizing definitions, labeling requirements, and safety thresholds across regulatory bodies is therefore essential, not only for consumer protection but also for enabling manufacturers to scale production with legal certainty. In parallel, food matrix adaptation requires more systematic investigation, given that the interactions between postbiotic components and matrix constituents remain inadequately characterized for most applications. Future research should accordingly adopt a matrix‐specific design approach, tailoring inactivation method, postbiotic form, and delivery strategy to the physicochemical demands of each food category. Scalability must be addressed concurrently with this laboratory‐scale innovation; bridging this gap will require closer collaboration between food process engineers and microbiologists to design scalable, energy‐efficient production systems without compromising bioactivity preservation. Taken together, these priorities, namely standardization, analytical validation, evidence based substantiation of health benefits, regulatory alignment, food matrix adaptation, and scalability, constitute an integrated roadmap for advancing postbiotics from a scientifically promising concept to a technologically mature and clinically substantiated category of functional food ingredients. Addressing them in a coordinated rather than fragmented manner will be essential for realizing the full potential of postbiotics within the global functional food industry.

## Author Contributions

**Tansu Taspinar:** conceptualization, investigation, funding acquisition, writing – original draft, visualization, writing – review and editing, software, resources. **Nuray Güzeler:** supervision, project administration.

## Funding

This work was supported by the Cukurova University Academic Research Projects Unit‐Grant ID FDK‐2021‐14080.

## Ethics Statement

This research is a literature‐based study that does not require any ethical documentation regarding the use of animal testing or human subjects.

## Conflicts of Interest

The authors declare no conflicts of interest.

## Data Availability

The authors have nothing to report.
